# Safety profiles of bone-conduction hearing implants revisited: A meta-analytic comparison adjusted for follow-up time

**DOI:** 10.1007/s00405-025-09502-w

**Published:** 2025-06-06

**Authors:** Marco Caversaccio, Wilhelm Wimmer, Annegret Hoch, Thomas Dejaco, Burkard Schwab

**Affiliations:** 1 Department of Otorhinolaryngology- Head and Neck Surgery, Inselspital, Bern University Hospital and University of Bern, Bern, Switzerland; 2 Department of Otorhinolaryngology, Klinikum Rechts Der Isar, Technical University of Munich, Munich, Germany; 3 MED-EL Elektromedizinische Geräte GmbH, Innsbruck, Austria; 4 HNO-Klinik, Helios Klinikum Hildesheim, Hildesheim, Germany

**Keywords:** Bone-conduction implant, Safety, Adverse event, Meta-regression

## Abstract

**Purpose:**

To estimate incidence rates of adverse events associated with bone-conduction hearing implants from primary literature and to compare rates among different technological designs.

**Methods:**

A systematic literature review and meta-regression was conducted to estimate incidence rates of minor and major complications and their consequences (i.e., revision surgery, explantation, re-implantation and becoming a non-user) while testing for effects of device design, age group, mean follow-up time and study type. These four designs of bone-conduction systems were included: 1) active transcutaneous with electromagnetic transducer (aBCIem), 2) active transcutaneous with piezoelectric transducer (aBCIpz), 3) passive transcutaneous (tBAHA), and 4) passive percutaneous (pBAHA).

**Results:**

The final dataset included 170 articles reporting on 6451 implantations and 1847 minor and 668 major events. Mean follow-up time was a significant predictor of incidence rates (p < 0.001), with lower rates reported in studies with longer follow-up times. After adjusting to the median follow-up time, the pooled incidence rate of minor complications was significantly lower in aBCIem (p < 0.05) compared to other designs. For both major events and revision surgery, pooled incidence rates were significantly higher in pBAHA compared to aBCIem (p < 0.001) and tBAHA (p < 0.001), but not compared to aBCIpz (Major: p = 0.197; Revision: p = 0.248). Becoming a non-user occurred significantly more frequently in tBAHA compared to other designs (p < 0.005). No statistically significant differences were found in rates of explantation and explantation with re-implantation.

**Conclusion:**

When comparing across multiple studies, adverse event rates should be adjusted for different lengths of follow-up. Synthesizing published evidence without considering follow-up time may lead to false conclusions.

**Supplementary Information:**

The online version contains supplementary material available at 10.1007/s00405-025-09502-w.

## Introduction

**Fig. 1:**
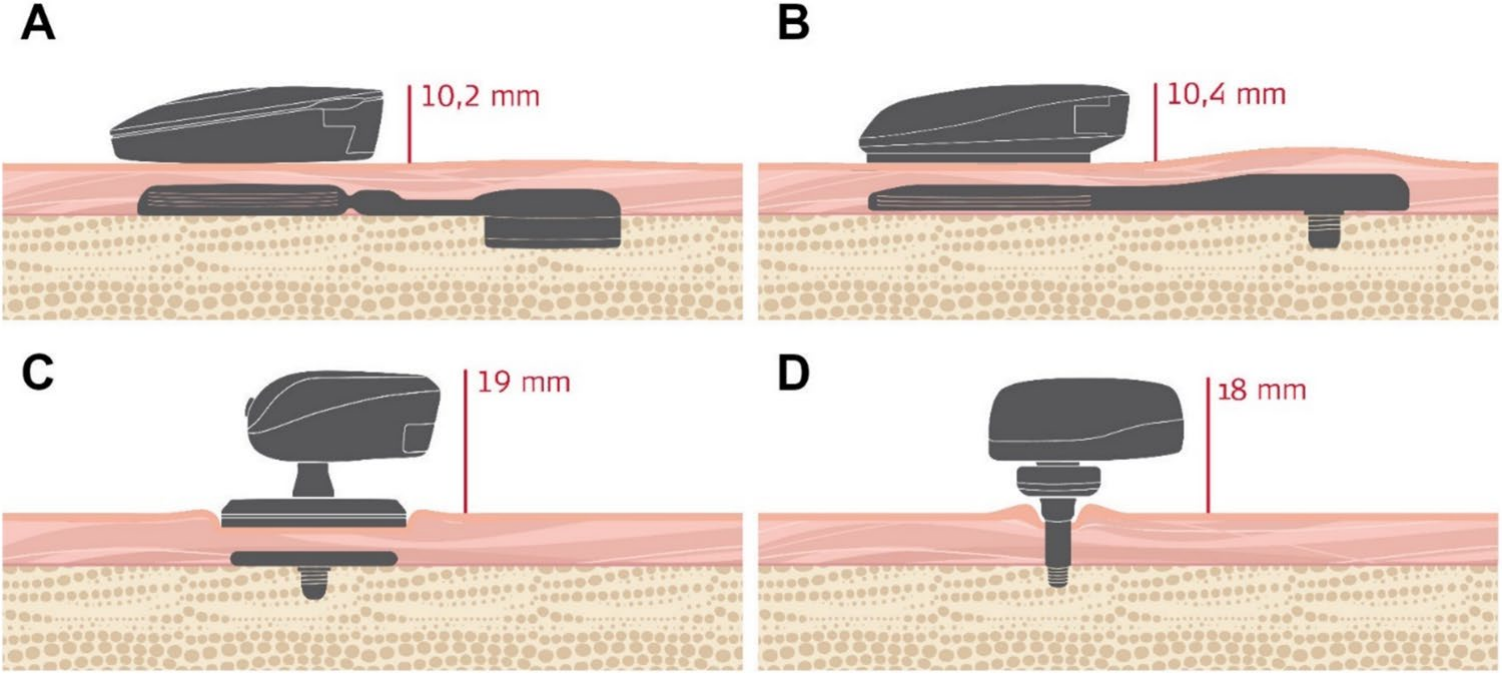
Four different designs of bone-conduction implants are currently available: Two designs include active implants (A, B), whereas the “BAHA” design includes passive implants (C, D). Active implants are transcutaneous by design. BAHA systems are either transcutaneous (C) or percutaneous (D)

**Fig. 2:**
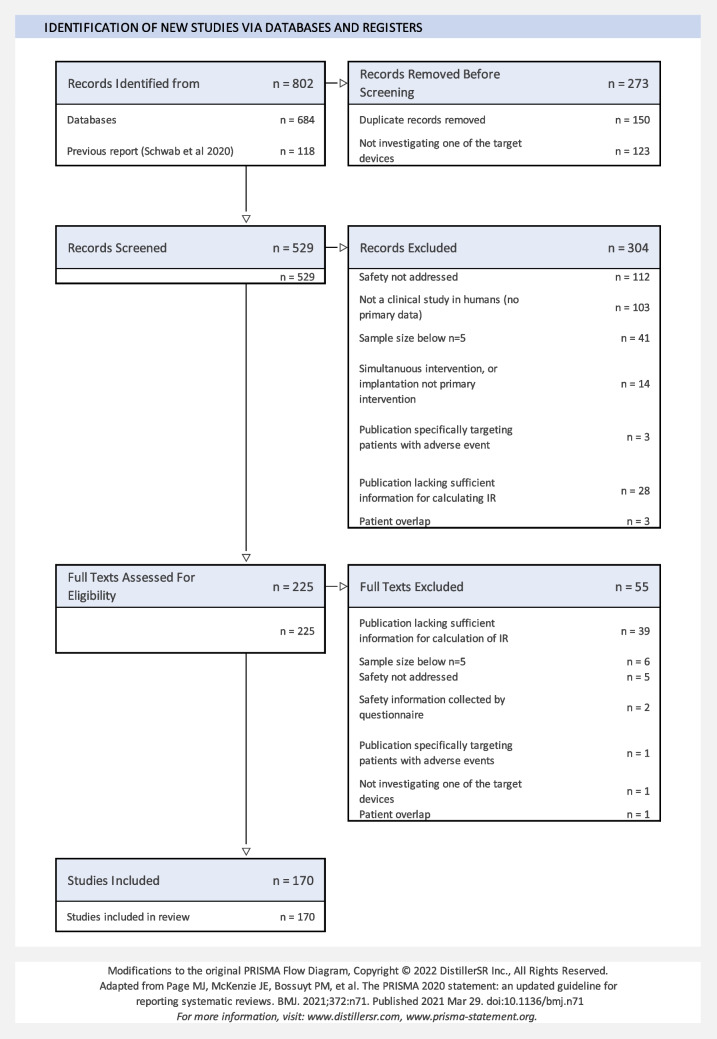
PRISMA2020 flowchart

**Fig. 3:**
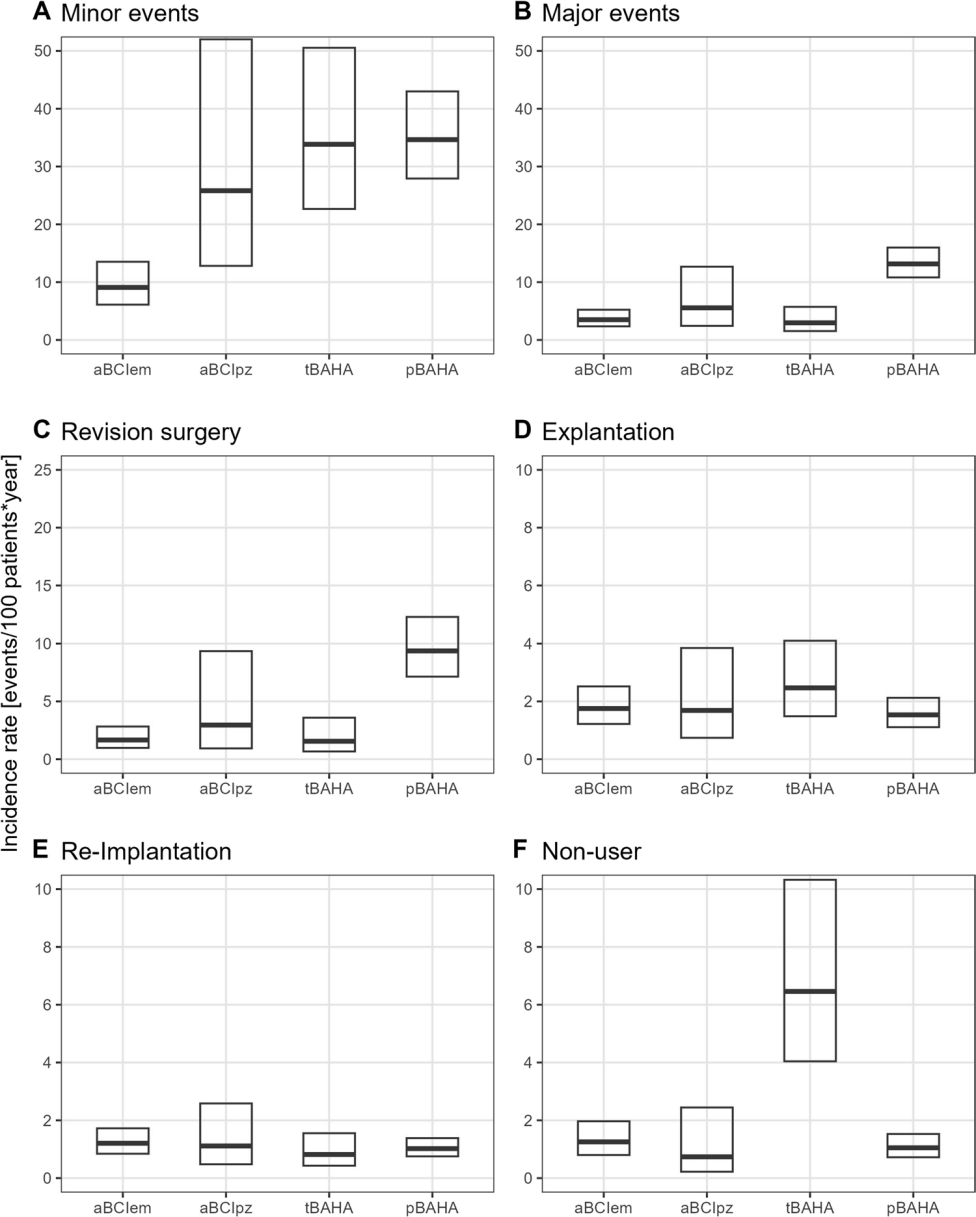
Pooled incidence rate of minor and major events, as well es revision surgery, explantation, explantation with re-implantation and non-user by device type adjusted to 12 months follow-up time

Bone-conduction hearing implants (BCIs) are a widely recognized and effective treatment option for patients suffering from conductive hearing loss (CHL), mixed hearing loss (MHL), or single-sided deafness (SSD). BCIs are surgically placed into the mastoid bone, where they transmit sound directly to the cochlea via vibrations, bypassing potentially impaired outer or middle ear structures. Currently, four different types of BCIs are available (Table 1), each based on differing technological design: 1) Active transcutaneous with electromagnetic transducer (Fig. 1A), 2) active transcutaneous with piezoelectric transducer (Fig. 1B), 3) passive transcutaneous (Fig. 1C), and 4) passive percutaneous (Fig. 1D). While most manufacturers now focus on developing active implants (aBCIs) instead of passive ones, all four design types continue to be offered to patients fulfilling the indication criteria. As medical devices of class II/IIb (USA/EU; passive devices) or class III (active devices), BCIs undergo ongoing safety evaluations by multiple stakeholders including manufacturers, notified bodies, healthcare providers and health technology assessment (HTA) agencies.

**Table 1:** Types of bone-conduction implants covered in this systematic review

Abbreviation	Design	Manufacturer/system name
aBCIem	active transcutaneous with electromagnetic transducer	MED-EL Bonebridge
aBCIpz	active transcutaneous with piezoelectric transducer	Cochlear OSIA
tBAHA	passive transcutaneous	Cochlear Attract, Medtronic (Sophono) Alpha
pBAHA	passive percutaneous	Cochlear Connect, Oticon Medical Ponto

While the published literature on the effectiveness of BCIs is extensive and the general safety of these devices is well-established, a dedicated comparison of risks for developing specific adverse events among different device types remains methodologically difficult due to heterogeneity in outcome definitions and follow-up times.

One reason for this challenge is the lack of comparability between results from different studies. For instance, several studies have used Kaplan–Meier estimators to assess implant survival in percutaneous [1, 2] and active transcutaneous implants [3]. However, the findings from these studies are hardly comparable due to different definitions of event types. Survival analysis is also not feasible in many cases due to the lack of patient-level data.

Most studies report an easy-to-calculate but simplified metric, the complication ratio, defined as the number of events relative to the number of implanted patients or ears. In these studies, the patient-time at risk is completely ignored. Consequently, most recent reviews focused on this outcome as well [4–7]. The few studies investigating complication ratios in a comparative design indicated that patients with active transcutaneous implants were at lower risk of developing post-operative complications compared to percutaneous implants [7]. Specifically, within the subgroup of transcutaneous implants, the active BCI with electromagnetic transducer (aBCIem) was associated with lower overall complication ratios compared to passive implants (pBAHA) [6].

In this systematic review, we evaluate the safety of BCIs with respect to incidence rate, defined as the number of adverse events relative to the total patient-time at risk [8–10]. To our knowledge, incidence rates of adverse events have not been reviewed systematically across multiple BCIs so far. Only one other review reported incidence rates for a subset of reviewed studies, focusing on one active BCI with electromagnetic transducer [11]. Here, we employ a classical meta-analytic approach and derive pooled estimates of incidence rates for different event types and across different device types. Given that original studies vary considerably in follow-up times, we use mixed-effects meta-regression models to examine the influence of confounding variables on these estimates and explicitly test hypotheses comparing different device designs. To include the entire body of published literature dating back to 1996, we extend the dataset compiled by Schwab et al. [7], including data published up to December 2023. Additionally, we also incorporate information on a relatively new piezoelectric active device and focus exclusively on BCIs.

## Materials and methods

### Systematic review

This review covers the literature on bone-conduction implants published between January 1996 and December 2023. It builds upon an earlier systematic review which included studies published up to 2016 [7]. To identify relevant articles, a new PUBMED search was conducted on June 3, 2024, using the following search string: (((OSIA OR BAHA OR BAHD OR Ponto OR Connect OR Attract OR Sophono OR Bonebridge) AND (bone-conduction implant))) AND ((hearing loss) OR (safety)) AND (2012:2023[pdat])) NOT (cochlear implant [MeSH Terms]). The search was restricted to articles in German and English, with no additional PUBMED filters applied.

All publications retrieved from PUBMED were imported into a web-based software for systematic reviews [12]. The PICOTS criteria used for screening are listed in Table 2. The number of excluded publications, along with the reason for exclusion, are summarized in a PRISMA2020 flowchart (Fig. 2). A total of 118 publications were carried forward from [7]. Combined with 52 newly identified articles, 170 publications were included in the review. Of these, 63 articles reported on active bone-conduction implants with electromagnetic transducers (aBCIem), 12 addressed active bone-conduction implants with piezoelectric transducers (aBCIpz), 79 covered passive percutaneous bone-conduction implants (pBAHA) and 24 focused on passive transcutaneous bone-conduction implants (tBAHA; see Online Resource 2 for full list of included publications).

**Table 2:** PICOTS definition

Population	Human subjects without restrictions on demographic parameters
Intervention	Implantation of any of the following bone-conduction devices: BONEBRIDGE, OSIA, BAHA Attract, BAHA Connect, Ponto, Sophono. No limitations on device generations
Comparator	Any of the devices mentioned under'Intervention'could be a comparator. Comparisons involving other devices were excluded
Outcome	All reported adverse events, excluding intra-operative complications Number of patients Mean follow-up time
Timing	Publications published between January 1, 2000 and December 31, 2023
Study Design	Studies reporting primary data for at least n = 5 ears. No additional restrictions

### Data extraction

We extracted demographic and event-related information from each study. Adverse events were defined as any untoward medical occurrences, unintended diseases or injuries related to the device or its implantation procedure. Events were classified as major we could conclude from the event and treatment description that it resulted in in-patient hospitalization or prolonged an existing hospitalization, in line with MEDDEV [13] and ISO14155 [14]. All other events were classified as minor. Additionally, we extracted common consequences of major adverse events, including revision surgery, explantation of the device, explantation followed by re-implantation of a new device, or the patient becoming a permanent non-user of the device.

### Quality assessment

All included publications were evaluated using the Oxford level of evidence for treatment benefit [15]. The overall quality and risk of bias were assessed with the Newcastle–Ottawa Scale (NOS) [16] for non-randomized cohort studies. NOS employs a star-based rating system across three domains: selection of the study groups, comparability of groups, ascertainment of exposure or outcome. Since two questions were not applicable to our safety outcome parameters, we conducted a modified assessment, allowing a maximum of 6 stars to be assigned to each study (see Table S1 in Online Resource 1).

### Data analysis

In a first step, a mixed-effects meta regression model was used to identify confounding variables with significant effect on the dependent variable, to be included in subsequent models. The confounders included were follow-up time in years (log transformed), age group (adult, children, or mixed), study type (prospective or retrospective) and device design (aBCIem, aBCIpz, tBAHA, pBAHA). Meta regression analysis revealed significant effects of both, device design and follow-up time on the yearly incidence, but no effect of age group and study type. Given the marked differences in follow-up durations across the different types of devices under review (see Figure S2 in Online Resource 1), follow-up time was determined to be a confounding factor when comparing adverse event incidence rates between device designs.

In a second step, the pooled incidence rates for each device design were estimated using separate mixed-effects models for each type of event (minor, major) and for each possible consequence of a major event (revision surgery, explantation, explantation with re-implantation). We also estimated a model for patients becoming non-users of their device. The regression equation is specified in Eq. 1 in Online Resource 1. Since mean F/U time had a significant effect in the first stage, the estimated incidence rates were adjusted to the median follow-up time of the sample (12 months) prior to comparing rates across device designs. These adjusted rates can be interpreted as yearly incidence rates, based on a patient population followed-up for 12 months.

Data was analyzed in R [17] within the RStudio [18] integrated development environment. The *metafor* package was used to conduct meta-regressions. Log-transformed incidence rates (“IRLN”), defined as the number of events divided by person-time at risk, were used as the effect size with an inverse-variance weight. For studies with zero events, a value of 0.1 events was assigned as a continuity correction to enable calculation of inverse variance weights. Mixed-effects meta-regression models were fit using the restricted maximum likelihood estimator (“REML”) for the between study variance τ^2^ [19]. Statistical heterogeneity was evaluated using I^2^,H^2^ and τ^2^ statistics [20], while potential publication bias was investigated via a formal test for funnel plot asymmetry. A sensitivity analysis was performed to examine the impact of continuity correction values other than 0.1 on the results. To improve the statistical properties of the test for individual model coefficients and the corresponding confidence intervals, we applied the Knapp and Hartung adjustment, which implies inference based on a t-distribution instead of a standard normal distribution [21]. To test for significant differences among devices, linear combinations of model coefficients were compared using a Wald-type test as implemented in the *metafor* R package [22].

## Results

### Descriptive statistics

The final dataset included 170 articles reporting on a total of 2559 adverse events among 6215 patients and 6451 implanted ears (Table 3). Patients were followed for an average of 25.5 months (median: 12.0 months), with substantial heterogeneity among devices types (see Figure S2 in Online Resource 1). Studies on pBAHA reported the longest follow-up periods, with a mean of 32.1 months and a median of 12.1 months. In contrast, studies on aBCIpz reported the shortest follow-up durations, with a mean of 11.0 months and a median of 6.0 months. Overall, the included studies documented 1888 minor events and 671 major events. Major events resulted either in revision surgery (N = 501), explantation of the device (N = 68) or explantation followed by re-implantation of a new device (N = 37). N = 42 patients became permanent non-users.

**Table 3:** Descriptive summary of information extracted from publications, by device design. N = sample size; Avg = average; F/U = follow-up time; rev = revision surgery; exp = explantation; reimpl = explantation with re-implantation; non = non-user; maj = major event; min = minor event

	Sample size			Follow-up time			Number of events					
Device	N studies	N patients	N ears	Median F/U [months]	Avg F/U [months]	Person-years	N rev	N exp	N reimpl	N nusr	N maj	N min
aBCIem	63	1367	1385	12.0	16.9	1925.4	8	12	10	8	29	84
aBCIpz	12	213	230	6.0	11.0	195.2	2	2	1	0	6	66
tBAHA	24	675	689	10.0	9.8	551.9	3	9	1	19	8	278
pBAHA	80	3870	4147	12.1	32.1	10,341.6	488	45	25	15	625	1419
Total	170	6125	6451	12.0	25.5	13,014.1	501	68	37	42	668	1847

The quality of the included studies was assessed with an average rating of 5.0 out of six possible stars on an adapted Newcastle–Ottawa quality assessment scale. All studies were rated as high quality across criteria 1 to 4 (“Representativeness of the exposed cohort”, “Ascertainment of exposure”, “Demonstration that the outcome was not present at the beginning of the study” and”Assessment of the outcome”). Fifty-nine percent of the studies had a follow-up time of 12 month or longer—which was considered as “Follow-up long enough for the safety outcomes to occur” (criterion 5) and were thus rated as high quality (i.e. evaluated with one star). However, only 43% of the included studies were rated as high quality regarding the “Adequacy of cohort follow-up” (criterion 6, which assesses wheter there were subjects lost to follow-up and whether this was likely to introduce bias). The rather low overall quality of the included studies was mainly due to variability in follow-up intervals among included patients or insufficient reporting of follow-up information (see Table S1 in Online Resource 1).

### Meta-analysis

The pooled incidence rates of minor events were estimated at 9.1 (95% CI:6.1–13.4) events per 100 patients per year for aBCIem, 25.8 (95% CI:13.0–51.4) for aBCIpz, 33.8 (95% CI:22.8–50.2) for tBAHA, and 34.7 (95% CI:28.0–42.9) for pBAHA (Fig. 3—A). Significfeant differences were found between aBCIem and each three other device designs (Table S2 in Online Resource 1).

When considering major events, the pooled incidence rates were estimated at 3.3 (95% CI:2.2–5.6) events per 100 patients per year for aBCIem, 5.6 (95% CI:2.1–14.5) for aBCIpz, 3.0 (95% CI:1.4–6.4) for tBAHA and 13.2 (95% CI:10.5–16.5) for pBAHA (Fig. 3—B). The rate for pBAHA was significantly higher compared with all other device designs (Table S3 in Online Resource 1).

The pooled incidence rates of revision surgery were estimated at 1.7 (95% CI:1.0–2.8) events per 100 patients per year for aBCIem, 3.0 (95% CI:0.9–9.3) for aBCIpz, 1.6 (95% CI:0.7–3.6) for tBAHA and 9.4 (95% CI:7.1–12.3) for pBAHA (Fig. 3—C). The rate for pBAHA was significantly higher compared with aBCIem and tBAHA (Table S4 in Online Resource 1).

Regarding explantation without re-implantation, the pooled incidence rates were estimated at 1.8 (95% CI:1.2–2.5) events per 100 patients per year for aBCIem, 1.7 (95% CI:0.7–3.8) for aBCIpz, 2.5 (95% CI:1.5–4.1) for tBAHA and 1.5 (95% CI:1.1–2.1) for pBAHA (Fig. 3—D). No statistically significant differences were found among the device types (Table S5 in Online Resources 1).

Similarly, pooled incidence rates of explantation with re-implantation were estimated at 1.2 (95% CI:0.8–1.7) events per 100 patients per year for aBCIem, 1.1 (95% CI:0.5–2.6) for aBCIpz, 0.8 (95% CI:0.4–1.6) for tBAHA and 0.8 (95% CI:0.4–1.6) for pBAHA (Fig. 3—E). Again, there were no statistically significant differences among the device types (Table S6 in Online Resources 1).

Finally, the pooled incidence rates of becoming a non-user were estimated at 1.3 (95% CI:0.8–2.0) events per 100 patients per year for aBCIem, 0.7 (95% CI:0.2–2.4) for aBCIpz, 6.5 (95% CI:4.0–10.3) for tBAHA and 1.1 (95% CI:0.7–1.5) for pBAHA (Fig. 3—F). In this instance, the rate for tBAHA was significantly higher compared with all other device designs (Table S7).

## Discussion

Implantable bone-conduction devices are a well-established treatment option for patients with CHL, MHL and SSD. Safety outcomes are continuously reported in studies evaluating implantable bone-conduction devices and the data may be used to monitor the safety profiles of BCIs. However, robust comparative analyses are lacking. To address this evidence gap, we conducted a systematic review and meta-analysis of the published literature on BCIs. Including device type and mean follow-up time as confounding variables, we formally tested for statistical differences among devices while controlling for different follow-up times. Our findings revealed significant differences in the risk of some post-operative complications among different device designs. In the absence of direct comparative evidence, our study represents an alternative approach to a quantitative, yet indirect comparison of adverse event incidence rates across different implantable BCIs.

### Transcutaneous vs. percutaneous design

Our findings support the superiority of transcutaneous over percutaneous designs in terms of overall safety profiles. Devices with transcutaneous designs (aBCIem, aBCIpz, tBAHA) exhibited significantly lower rates of major complications and revision surgeries compared with percutaneous designs (pBAHA). Regarding minor events, percutaneous implants demonstrated a significantly higher risk of complications compared to aBCIem, but not compared to the other device types (tBAHA and aBCIpz). From this finding we conclude that transcutaneous designs significantly reduce the risk for major, but not for minor adverse events. The different risks in minor events could be explained by other attributes like fixation with one vs two screws, different implant shapes or even differences in surgical procedures. Whether the “active implant” attribute (aBCIem and aBCIpz) has a significant effect is not evident from our data mainly due to low sample size and high variability in the aBCIpz device group.

An interesting aspect was observed in the risk for permanent non-use of the device. While patients who discontinued using their device typically did not require explanation—and non-use is not considered a serious adverse event—permanent non-use is a critical outcome as affected patients no longer benefit from the actual treatment. Based on our results, the risk of becoming a non-user was significantly elevated (6.5 in 100 patients per year) for passive transcutaneous implants (tBAHA) compared to other device types. Unfortunately, the reason for stopping device usage was only stated in 3 out of 42 reports. These included permanent removal of abutment (pBAHA; N = 1), skin erythema and severe pain (tBAHA; N = 1) and discomfort and aesthetics (tBAHA; N = 1). We did not include secondary (sleeper) BAHA placements or dysfunctional primary placements as non-users. These were not included at all in our analysis. In the absence of more detailed information, we hypothesize that the elevated risk for stopping device usage in the tBAHA group may be due to a combination of effects, mainly high risk for skin-related minor events and lower audiological effectiveness due to a dampening effect of the skin layer (i.e. indirect stimulation of the mastoid bone).

### Active BCIs

Active transcutaneous BCIs are state-of-the-art in the field of BCIs and two different designs, i.e., involving electromagnetic or piezoelectric transducers, are currently available. Therefore, a comparative discussion is of particular interest to the scientific community. Our analysis included data from 1385 implantations with a mean follow-up time of 16.9 months in the aBCIem group, and 230 implantations with a mean follow-up time of 11 months in the aBCIpz group. A third device (also to be placed in the aBCIem group) is currently entering the market but was not included in this analysis due to a very limited amount of available data.

Studies using the aBCIem device reported significantly lower risks of minor events (on average 9.1 events per 100 patients per year) compared to 25.8 events per 100 patients per year in studies using the aBCIpz device. Our analysis was not able to identify unique factors responsible for the observed difference. However, most minor complications were skin-related (i.e., swelling, pain, redness or transient wound infections) for both active implants. A potential explanation for the risk difference among these devices could lie in the strikingly different shapes of transducers. The transducer of aBCIem devices has a smaller diameter and a sagittal profile that matches the curvature of the mastoid bone. Also, it is recessed in a bone bed during surgery, thus minimizing skin displacement. In contrast, the transducer of the aBCIpz device has a larger diameter and a flat sagittal profile. Because the piezoelectric transducer is not allowed to be in contact with underlying bone, and because cortical bone is needed for proper osseointegration of the central screw, the protruding transducer results in substantial displacement of skin layers above the implant compared to the aBCIem device. Altogether, these factors may lead to more frequent skin-related events in the aBCIpz group, prompting many authors to explore alternative surgical techniques [23–27]. Other potential reasons include different surgical procedures involving specific incision techniques, skin thinning or other, hitherto unknown factors. However, the data extracted in this systematic review did not provide enough detail to formally test potential effects of these parameters.

Concerning major events, studies in the aBCIem group found on average lower risks (3.5 events per 100 patients per year) than those in the aBCIpz group (5.6 events per 100 patients per year), but this difference was not statistically significant. Likewise, no significant differences were found in the risk for explantation, revision surgery, re-implantation or permanent non-use. Again, the smaller sample size translated to wider confidence intervals in the aBCIpz group. As the published body of evidence for the aBCIpz system will continue to grow, the ability to detect significant differences in safety outcomes may improve accordingly. Currently, our data does not support the hypothesis of lower impact resistance of the single point fixation (aBCIpz) compared to the two-point fixation design in the aBCIem group.

Our findings largely match the results of Schwab et al. [7] which highlighted lower complication ratios for minor events for the Bonebridge (aBCIem) compared to transcutaneous and percutaneous BAHAs. Schwab et al. also reported a higher incidence of skin-related complications, both in frequency and severity, associated with percutaneous devices.

Only one other review has reported yearly rates of adverse events [11], though for a single device only. The meta-analysis by Magele et al. included 25 publications (286 Bonebridge implantations) and estimated a minor complication rate of 10 events per 100 patients per year^1^which closely aligns with the 9 events per 100 patients per year found in our analysis. However, the major complication rate reported in that review was much lower at 0.7 events per 100 patients per year^2^compared with 3.5 events per 100 patients per year found in our analysis. Our current estimate for the Bonebridge device is based on a significantly larger body of evidence, making it more robust and reliable compared to Magele et al. 2019.

### Methodological aspects

The few comparative reviews on safety with BCIs have typically looked at complication ratios instead of incidence rates [6, 7]. Importantly, ratios should only be compared across studies with equal follow-up times, as they do not include any information on the time patients have been at risk for developing any event. Consequently, our results should only be compared to studies reporting complication ratios calculated from studies with a follow-up period of roughly 1 year.

The strength of our approach lies in the ability to include and compare data from all studies with varying follow-up times by explicitly adjusting for patient-time at risk. To our knowledge, this approach has not been applied so far. One possible reason for that is that most studies and guidelines such as the Cochrane Handbook for Systematic Reviews of Interventions [28] focus on highly controlled settings, where outcomes are compared between treatment and control arms with mostly equal follow-up times. Also, commonly derived measures such as risk ratios or hazard ratios are less prone to bias due to differences in follow-up times. For incidence rates in single-armed trials, however, including follow-up time as a confounder seems necessary when comparing interventions across studies with systematic differences in follow-up time. Furthermore, the yearly rates derived from this analysis provide a standardized metric for use in economic models of costs and benefits (e.g. Markov-models).

### Limitations

We detected significant funnel plot asymmetry in our meta-analyses (see Figure S3 in Online Resource 1), mainly introduced by studies with zero events and the continuity correction that was necessary to include those studies to the meta-analysis. This is an inherent drawback of using the classic inverse variance method for meta-analysis of incidence rates, since sampling variances need to be calculated for all studies, including those reporting zero events. In a robustness analysis, we examined how the pooled effects were influenced by the choice of the continuity correction (and the corresponding variance). While the continuity correction values significantly impacted the absolute values of the pooled incidence rates, it did not affect the statistical significance of the comparisons between device designs.

An alternative approach for count data would be to estimate a general linear mixed model (GLMM) based on a Poisson distribution. Such a model would not require the assumption of normally distributed values within a study. A “Poisson-normal” model was applied by Niël-Weise et al. 2008 [29] to analyze incidence rates across multiple studies. While the inclusion of follow-up time as an offset-term is feasible, estimating incidence rate differences based on a Poisson distribution becomes methodologically challenging when extending beyond a two-group comparison and adjusting for covariates, as it was required for our analysis of four device types [30]. An alternative that allows for the use of Poisson regression models and the inclusion of moderator variables would be an individual patient data meta-analysis [31]. However, this would imply to restrict the analysis to a small subset of studies for which raw data is available. In conclusion, despite the limitations mentioned above, our meta-analytic approach proved to be suitable for synthesizing the entire body of evidence across studies with different device designs.

We want to emphasize that our study does not allow for conclusions regarding the specific timing of events. To address this, patient-level data from large study cohorts or registries would be required. Unfortunately, the existing body of evidence is predominantly composed of small-to-moderately-sampled studies with heterogenous designs, varying follow-up durations, and inconsistent quality. The reporting quality of the included studies was a notable limitation. A total of 67 studies had to be excluded due to insufficient information for calculating incidence rates. In most cases, the exclusion was due to the lack of clarity regarding the timeframe over which the reported events occurred.

## Supplementary Information

Below is the link to the electronic supplementary material.

Supplementary file1 (DOCX 747 KB)

Supplementary file2 (DOCX 50.8 KB)

## Footnotes

1 Reported as 1 in 10 minor adverse events per year of follow-up.

2 Reported as 7 major adverse events in 1000 subjects per follow-up year.
