# Correction: Safety profiles of bone-conduction hearing implants revisited: A meta-analytic comparison adjusted for follow-up time

**DOI:** 10.1007/s00405-025-09623-2

**Published:** 2025-08-29

**Authors:** Marco Caversaccio, Wilhelm Wimmer, Annegret Hoch, Thomas Dejaco, Burkard Schwab

**Affiliations:** 1 Department of Otorhinolaryngology‑ Head and Neck Surgery, Inselspital, Bern University Hospital and University of Bern, Bern, Switzerland; 2 Department of Otorhinolaryngology, Klinikum Rechts Der Isar, Technical University of Munich, Munich, Germany; 3 MED-EL Elektromedizinische Geräte GmbH, Innsbruck, Austria; 4 HNO-Klinik, Helios Klinikum Hildesheim, Hildesheim, Germany

**Correction:** **European Archives of Oto-Rhino-Laryngology**

10.1007/s00405-025-09502-w

In this article, Fig(3) appeared incorrectly and have now been corrected in the original publication. For completeness and transparency, the old incorrect and correct versions are displayed below.

**Incorrect Version**


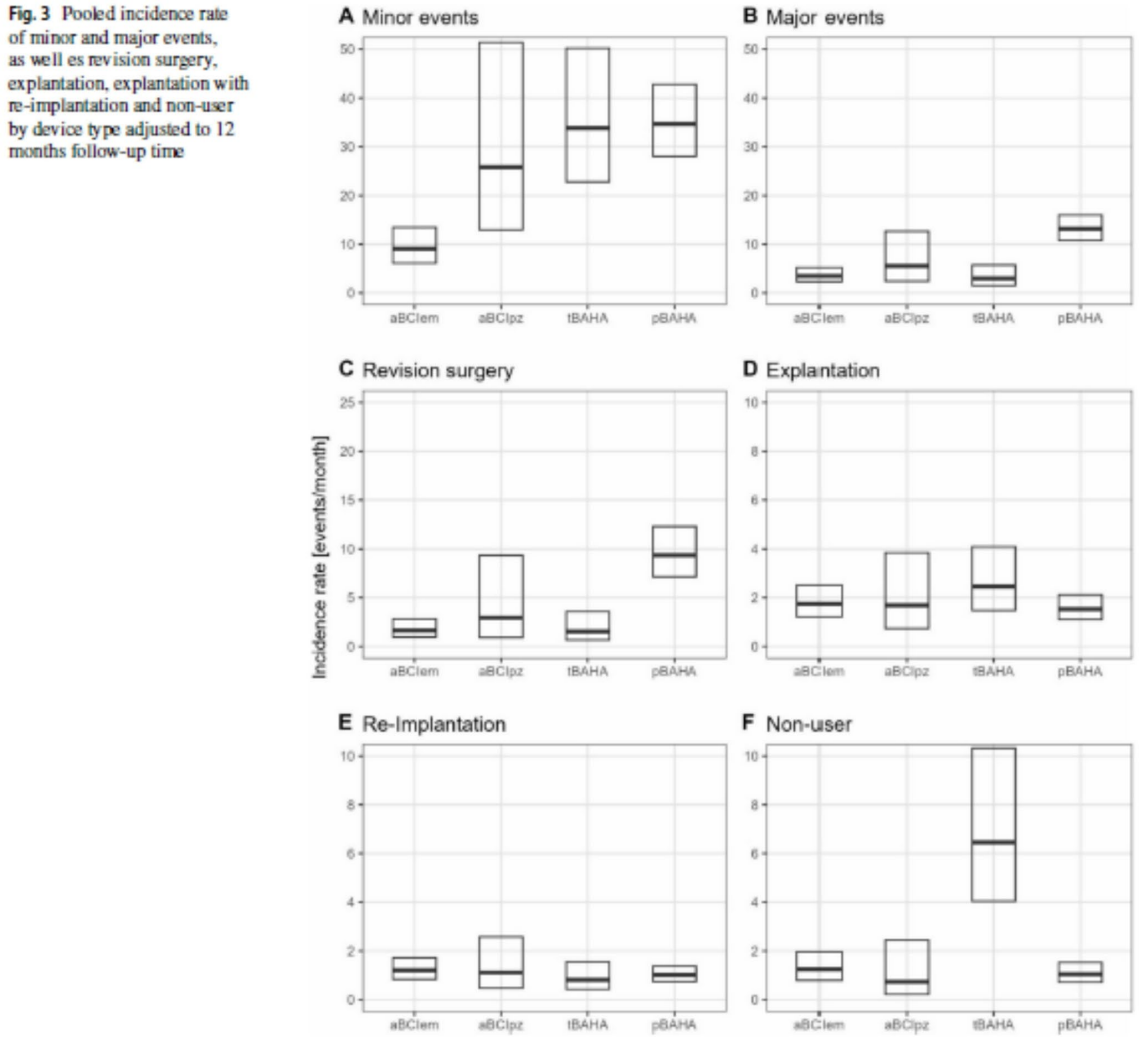


**Correct version**

**Fig. 3:**
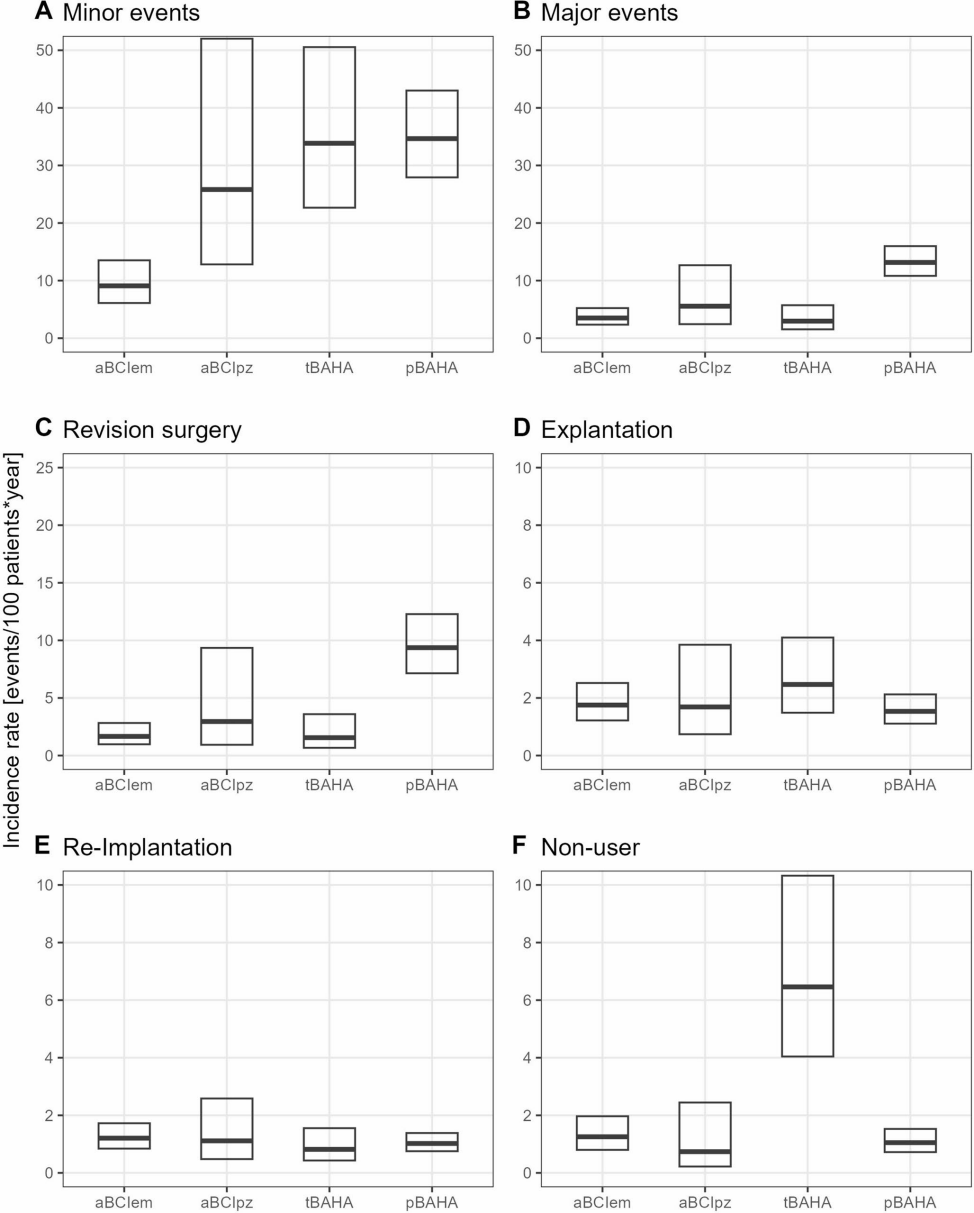
Pooled incidence rate of minor and major events, as well es revision surgery, explantation, explantation with re-implantation and non-user by device type adjusted to 12 months follow-up time

The original article has been corrected.

