# Supplementary material for: Safety profiles of bone-conduction hearing implants revisited: A meta-analytic comparison adjusted for follow-up time
Source: Eur Arch Otorhinolaryngol. 2025 Jun 6;282(11):5529–38. doi: 10.1007/s00405-025-09502-w (PMC12605608; doi:10.1007/s00405-025-09502-w)
Supplement: Supplementary file 1 — Supplementary file1 (DOCX 747 KB) [file 405_2025_9502_MOESM1_ESM.docx]

**Online Resource 1**

*Quality assessment*

We used the Newcastle-Ottawa quality assessment scale for cohort studies. For the safety evaluation, there is no study design with a control or non-exposed cohort feasible (even though for the audiological assessment, patients serve as their own controls in a before-after implantation/unaided-aided comparison). As several questions were not applicable to safety, we conducted a modified assessment where a maximum of 6 stars could be awarded for each study.

Table S1: Assessment of reporting quality based on the Newcastle-Ottawa Scale for cohort studies

| Section | Question | Explanation | Assessment |
| --- | --- | --- | --- |
| Selection | Representativeness of the exposed cohort | All selected studies were truly or somewhat representative of the exposed cohort and described the derivation of the cohort | 100 % of studies with one star |
|  | Selection of the non-exposed cohort | For the safety evaluation, there is no control group, so there is no non-exposed cohort | Not applicable |
|  | Ascertainment of exposure | All outcomes were reported based on secured records | 100 % of studies with one star |
|  | Demonstration that outcome of interest was not present at start of study | No need to demonstrate for adverse events | 100 % of studies with one star |
| Comparability | Comparability of cohorts on the basis of the design or analysis | For the safety evaluation, there is no control group | Not applicable |
| Outcome | Assessment of outcome | All outcomes were assessed via record linkage | 100 % of studies with one star |
|  | Was follow-up long enough for outcomes to occur | As long enough follow-up period, we considered 12 months or longer | 59 % of studies with one star |
|  | Adequacy of follow up of cohorts | Chart reviews typically had different follow-up times for each patient | 43 % of studies with one star |

*Meta-regression*

The variable to predict in our regression model is the observed effect size $\hat{\theta}_{k}$of study *k*

$\hat{\theta}_{k}=\theta+\beta_{1}{FU}_{k}+\beta_{2}{DT}_{k}+\epsilon_{k}+\zeta_{k}$ (1)

 with the intercept $\theta,$ the follow-up time of study ${FU}_{k}$ and a categorial variable ${DT}_{k}$specifying the device type. $\epsilon_{k}$ denotes the sampling error and $\zeta_{k}$ the between-study heterogeneity $.$ While fixed effects meta-regression assumes that sampling error is the only source of variance in overall study effect sizes, in our mixed effects model we also account for a random component$\zeta_{k}$ (Borenstein et al 2023).

We found significant differences in incidence rates between device designs (aBCIem vs. aBCIpz: p=0.007, aBCIem vs. tBAHA: p<0.0001, aBCIem vs. pBAHA: p<0.0001). This finding indicates that the device designs are associated with different risk profiles of adverse events.

The regression model further revealed a significant negative effect (p<0.0001) of the follow-up time on the yearly incidence of adverse events. The longer the follow-up time of the study, the lower is the yearly incidence of events. Hence, studies with a shorter follow-up time tend to report higher yearly incidence rates than studies following patients over a longer time horizon. This pattern is related to the timing of events: most adverse events with bone conduction devices are related to the implantation procedure and occur within the first 12 months after implantation.





Figure S1: Mixed-effects model – regression of log incidence rate on log follow-up time


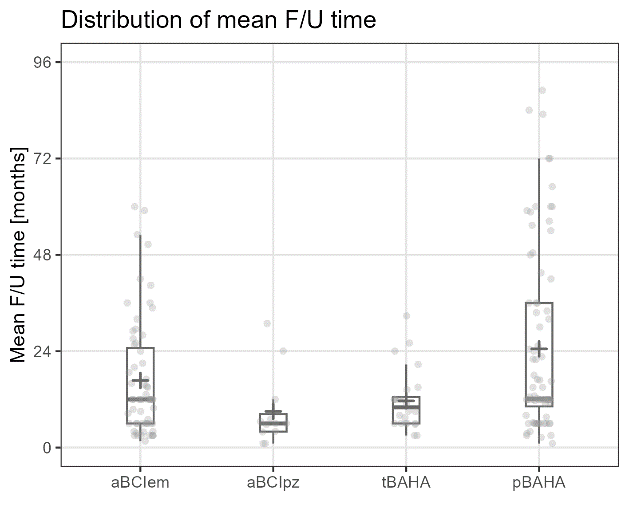


Figure S2: Distribution of mean follow-up times reported in single studies, by device design.

Table S2: Pairwise comparison of minor incidence rates across device designs. Each line corresponds to one comparison pair. se=standard error, zval=value of the z statistic, pval=p value.

| **hypotheses** | **estimate** | **se** | **zval** | **pval** | **adjusted pval** |
| --- | --- | --- | --- | --- | --- |
| aBCIem vs. aBCIpz | 2.841 | 1.494 | 2.601 | **0.009** | **0.043** |
| aBCIem vs. tBAHA | 3.724 | 1.327 | 4.649 | **<0.001** | **<0.001** |
| aBCIem vs. pBAHA | 3.813 | 1.256 | 5.867 | **<0.001** | **<0.001** |
| aBCIpz vs. tBAHA | 1.311 | 1.493 | 0.676 | 0.499 | 1.000 |
| aBCIpz vs. pBAHA | 1.342 | 1.453 | 0.788 | 0.431 | 1.000 |
| tBAHA vs. pBAHA | 1.024 | 1.259 | 0.103 | 0.918 | 1.000 |

Note: Pairwise comparison of device-specific incidence rate after adjustment to the median, i.e. 1 year of follow-up. Each line corresponds to one comparison-pair.

Table S3: Pairwise comparison of major incidence rates across device designs. Each line corresponds to one comparison pair. se=standard error, zval=value of the z statistic, pval=p value.

| **hypotheses** | **estimate** | **se** | **zval** | **pval** | **adj. pval** |
| --- | --- | --- | --- | --- | --- |
| aBCIem vs. aBCIpz | 1.584 | 1.730 | 0.840 | 0.401 | 0.720 |
| aBCIem vs. tBAHA | 0.845 | 1.582 | -0.366 | 0.714 | 0.720 |
| aBCIem vs. pBAHA | 3.750 | 1.299 | 5.052 | **<0.001** | **<0.001** |
| aBCIpz vs. tBAHA | 0.533 | 1.871 | -1.003 | 0.316 | 0.720 |
| aBCIpz vs. pBAHA | 2.367 | 1.667 | 1.685 | 0.092 | 0.197 |
| tBAHA vs. pBAHA | 4.437 | 1.507 | 3.632 | **<0.001** | **<0.001** |

Note: Pairwise comparison of device-specific incidence rate after adjustment to the median, i.e. 1 year of follow-up. Each line corresponds to one comparison-pair.

Table S4: Pairwise comparison of revision surgery incidence rates across device designs. Each line corresponds to one comparison pair. se=standard error, zval=value of the z statistic, pval=p value.

| **hypotheses** | **estimate** | **se** | **zval** | **pval** | **adj. pval** |
| --- | --- | --- | --- | --- | --- |
| aBCIem vs. aBCIpz | 1.777 | 1.905 | 0.892 | 0.374 | 1.000 |
| aBCIem vs. tBAHA | 0.933 | 1.654 | -0.137 | 0.891 | 1.000 |
| aBCIem vs. pBAHA | 5.632 | 1.350 | 5.762 | **<0.001** | **<0.001** |
| aBCIpz vs. tBAHA | 0.525 | 2.055 | -0.894 | 0.373 | 1.000 |
| aBCIpz vs. pBAHA | 3.170 | 1.848 | 1.879 | 0.062 | 0.248 |
| tBAHA vs. pBAHA | 6.034 | 1.565 | 4.014 | **<0.001** | **<0.001** |

Note: Pairwise comparison of device-specific incidence rate after adjustment to the median, i.e. 1 year of follow-up. Each line corresponds to one comparison-pair.

Table S5: Pairwise comparison of explantation incidence rates across device designs. Each line corresponds to one comparison pair. se=standard error, zval=value of the z statistic, pval=p value.

| **hypotheses** | **estimate** | **se** | **zval** | **pval** | **adj. pval** |
| --- | --- | --- | --- | --- | --- |
| aBCIem vs. aBCIpz | 0.963 | 1.579 | -0.083 | 0.934 | 1.000 |
| aBCIem vs. tBAHA | 1.408 | 1.373 | 1.078 | 0.282 | 1.000 |
| aBCIem vs. pBAHA | 0.875 | 1.267 | -0.562 | 0.575 | 1.000 |
| aBCIpz vs. tBAHA | 1.462 | 1.632 | 0.776 | 0.439 | 1.000 |
| aBCIpz vs. pBAHA | 0.909 | 1.570 | -0.211 | 0.833 | 1.000 |
| tBAHA vs. pBAHA | 0.622 | 1.363 | -1.534 | 0.127 | 0.761 |

Note: Pairwise comparison of device-specific incidence rate after adjustment to the median, i.e. 1 year of follow-up. Each line corresponds to one comparison-pair.

Table S6: Pairwise comparison of explantation with re-implantation incidence rates across device designs. Each line corresponds to one comparison pair. se=standard error, zval=value of the z statistic, pval=p value.

| **hypotheses** | **estimate** | **se** | **zval** | **pval** | **adj. pval** |
| --- | --- | --- | --- | --- | --- |
| aBCIem vs. aBCIpz | 0.923 | 1.590 | -0.173 | 0.862 | 1.000 |
| aBCIem vs. tBAHA | 0.678 | 1.451 | -1.044 | 0.298 | 1.000 |
| aBCIem vs. pBAHA | 0.847 | 1.268 | -0.701 | 0.484 | 1.000 |
| aBCIpz vs. tBAHA | 0.735 | 1.707 | -0.576 | 0.566 | 1.000 |
| aBCIpz vs. pBAHA | 0.918 | 1.574 | -0.190 | 0.850 | 1.000 |
| tBAHA vs. pBAHA | 1.249 | 1.433 | 0.617 | 0.538 | 1.000 |

Note: Pairwise comparison of device-specific incidence rate after adjustment to the median, i.e. 1 year of follow-up. Each line corresponds to one comparison-pair.

Table S7: Pairwise comparison of non-user incidence rates across device designs. Each line corresponds to one comparison pair. se=standard error, zval=value of the z statistic, pval=p value.

| **hypotheses** | **estimate** | **se** | **zval** | **pval** | **adj. pval** |
| --- | --- | --- | --- | --- | --- |
| aBCIem vs. aBCIpz | 0.587 | 1.915 | -0.819 | 0.414 | 1.000 |
| aBCIem vs. tBAHA | 5.148 | 1.390 | 4.976 | **<0.001** | **<0.001** |
| aBCIem vs. pBAHA | 0.837 | 1.344 | -0.600 | 0.549 | 1.000 |
| aBCIpz vs. tBAHA | 8.765 | 1.919 | 3.331 | **0.001** | **0.004** |
| aBCIpz vs. pBAHA | 1.426 | 1.908 | 0.549 | 0.584 | 1.000 |
| tBAHA vs. pBAHA | 0.163 | 1.356 | -5.962 | **<0.001** | **<0.001** |

Note: Pairwise comparison of device-specific incidence rate after adjustment to the median, i.e. 1 year of follow-up. Each line corresponds to one comparison-pair.

The heterogeneity in our models differs substantially between the six models. Interestingly, we observe a considerable heterogeneity (indicated by an I^2^ above 60 %) for minor and major events and revison surgery, and substantially lower heterogeneity for explantation, explantation with re-implantation and non-user (I^2^ below 25 %). A very low heterogeneity is found for those types of events, for which most studies report zero events.

Table S8: Measures of heterogeneity for final meta-analytic models of each event category.

|  | I^2^ | H^2^ | τ^2^ |
| --- | --- | --- | --- |
| Minor events | 86.1 | 7.2 | 0.64 |
| Major events | 65.6 | 2.9 | 0.53 |
| Revision surgery | 69.7 | 3.3 | 0.89 |
| Explantation | 14.6 | 1.2 | 0.43 |
| Explantation with re-implantation | 19.3 | 1.2 | 0.95 |
| Non-user | 21.6 | 1.3 | 0.96 |


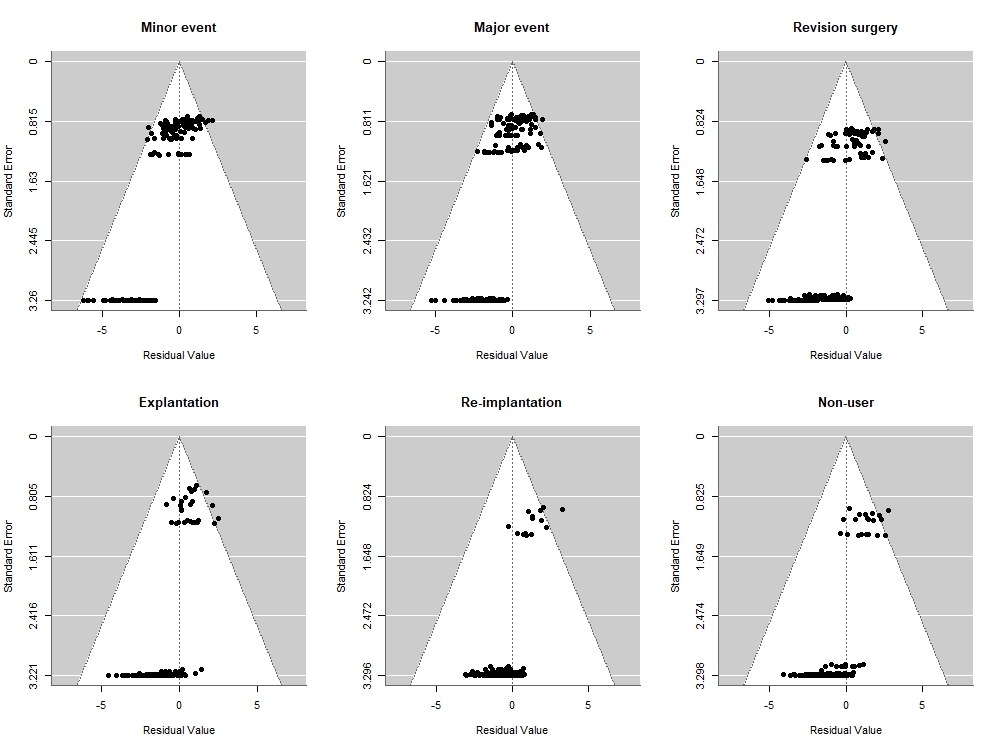


Figure S3. Funnel plot for final meta-analytic models of each event category.
