# Supplementary material for: Safety profiles of bone-conduction hearing implants revisited: A meta-analytic comparison adjusted for follow-up time
Source: Eur Arch Otorhinolaryngol. 2025 Jun 6;282(11):5529–38. doi: 10.1007/s00405-025-09502-w (PMC12605608; doi:10.1007/s00405-025-09502-w)
Supplement: Supplementary file 2 — Supplementary file2 (DOCX 50.8 KB) [file 405_2025_9502_MOESM2_ESM.docx]

**Online Resource 2**

Included publications investigating aBCIem devices:

^1–63^

Included publications investigating aBCIpz devices:

^39,64–74^

Included publications investigating tBAHA devices:

^19,42,75–96^

Included publications investigating pBAHA devices:

^16,19,83,85,91,97–170^

1. Alzhrani F. Objective and subjective results of the Bonebridge transcutaneous active direct-drive bone conduction hearing implant. *Saudi Med J*. 2019;40(8):797-801. doi:10.15537/smj.2019.8.24383

2. Baumgartner WD, Hamzavi JS, Boheim K, et al. A New Transcutaneous Bone Conduction Hearing Implant: Short-term Safety and Efficacy in Children. *Otol Neurotol*. 2016;37(6):713-720. doi:10.1097/mao.0000000000001038

3. Brkic FF, Baumgartner WD, Schlott M, et al. Experience With the New Active Transcutaneous Bone-Conduction Implant With      Smaller Dimensions. *Otolaryngol Head Neck Surg*. 2023;169:615-621. doi:10.1002/ohn.269

4. Brkic FF, Riss D, Scheuba K, et al. Medical, Technical and Audiological Outcomes of Hearing Rehabilitation with the Bonebridge Transcutaneous Bone-Conduction Implant: A Single-Center Experience. *J Clin Med*. 2019;8(10). doi:10.3390/jcm8101614

5. Canale A, Boggio V, Albera A, et al. A new bone conduction hearing aid to predict hearing outcome with an active implanted device. *Eur Arch Otorhinolaryngol*. 2019;276(8):2165-2170. doi:10.1007/s00405-019-05450-4

6. Canale A, Ndrev D, Sapino S, Bianchi C, Bordino V, Albera A. Speech in Noise With Bilateral Active Bone Conduction Implant for Conductive and Mixed Hearing Loss. *Otol Neurotol*. 2022;43(9):1000-1004. doi:10.1097/mao.0000000000003671

7. Canzi P, Avato I, Beltrame M, et al. Retrosigmoidal placement of an active transcutaneous bone conduction implant: surgical and audiological perspectives in a multicentre study. *Acta Otorhinolaryngol Ital*. 2021;41(1):91-99. doi:10.14639/0392-100x-n0609

8. Carnevale C, Til-Perez G, Arancibia-Tagle DJ, Tomas-Barberan MD, Sarria-Echegaray PL. Hearing outcomes of the active bone conduction system Bonebridge((R)) in conductive or mixed hearing loss. *Acta Otorrinolaringol Esp (Engl Ed)*. 2018;70:80-88. doi:10.1016/j.otorri.2018.02.006

9. Carnevale C, Tomas-Barberan M, Til-Perez G, Sarria-Echegaray P. The Bonebridge active bone conduction system: a fast and safe technique for a middle fossa approach. *J Laryngol Otol*. 2019;133(4):344-347. doi:10.1017/s0022215119000501

10. Chan KC, Wallace CG, Ho VWY, Wu CM, Chen HY, Chen ZC. Simultaneous auricular reconstruction and transcutaneous bone conduction device      implantation in patients with microtia. *J Formos Med Assoc*. 2019;118:1202-1210. doi:10.1016/j.jfma.2019.01.013

11. Cheon JH, Lee HC, Im GJ, Park JY, Park C. Safety and efficacy of transcutaneous bone conduction implant surgery for hearing      improvement in microtia patients with bilateral hearing impairment. *Arch Plast Surg*. 2019;46:525-534. doi:10.5999/aps.2019.00661

12. Cywka KB, Skarzynski PH, Krol B, Hatzopoulos S, Skarzynski H. Evaluation of the Bonebridge BCI 602 active bone conductive implant in adults: efficacy and stability of audiological, surgical, and functional outcomes. *Eur Arch Otorhinolaryngol*. 2022;279(7):3525-3534. doi:10.1007/s00405-022-07265-2

13. Der C, Bravo-Torres S, Pons N. Active Transcutaneous Bone Conduction Implant: Middle Fossa Placement Technique in Children With Bilateral Microtia and External Auditory Canal Atresia. *Otol Neurotol*. 2018;39(5):e342-e348. doi:10.1097/mao.0000000000001809

14. Deun LV, Voecht KD, Desloovere C, Verhaert N. Safety and efficacy of the Bonebridge bone conduction implant: a comparative study. *B-ENT*. 2020;16(1):9-14. doi:10.5152/b-ent.2020.19123

15. Eberhard KE, Olsen SO, Miyazaki H, Bille M, Caye-Thomasen P. Objective and Subjective Outcome of a New Transcutaneous Bone Conduction Hearing Device: Half-year Follow-up of the First 12 Nordic Implantations. *Otol Neurotol*. 2016;37(3):267-275. doi:10.1097/mao.0000000000000969

16. Fan X, Yang T, Niu X, Wang Y, Fan Y, Chen X. Long-term Outcomes of Bone Conduction Hearing Implants in Patients With Bilateral Microtia-atresia. *Otol Neurotol*. 2019;40(8):998-1005. doi:10.1097/mao.0000000000002370

17. Gao M, Zhao C, Yang J, et al. Bone-conduction hearing aid is effective in congenital oval window atresia. *Acta Otolaryngol*. 2021;141(4):321-327. doi:10.1080/00016489.2020.1866211

18. Garcier M, Lavedrine A, Gagneux C, Eluecque T, Grayeli AB. Bone-Anchored and Closed Skin Bonebridge Implant in Adults: Hearing Performances and Quality of Life. *Audiol Neurootol*. 2021;26(5):310-316. doi:10.1159/000512496

19. Han JJ, Park HR, Song JJ, Koo JW, Choi BY. A comparison study of audiological outcome and compliance of bone conduction implantable hearing implants. *Eur Arch Otorhinolaryngol*. 2020;277(11):3003-3012. doi:10.1007/s00405-020-06025-4

20. Harris MK, Kaul VF, Bergman M, Dodson EE, Ren Y, Adunka OF. Outcomes After Transcutaneous Bone-Conduction Implantation in Adults and Children. *Otol Neurotol*. 2023;44(4):317-323. doi:10.1097/mao.0000000000003821

21. Huber AM, Strauchmann B, Caversaccio MD, et al. Multicenter Results With an Active Transcutaneous Bone Conduction Implant in Patients With Single-sided Deafness. *Otol Neurotol*. 2022;43(2):227-235. doi:10.1097/mao.0000000000003418

22. Hundertpfund J, Meyer JE, Ovari A. Long-term audiological benefit with an active transcutaneous bone-conduction device: a retrospective cohort analysis. *Eur Arch Otorhinolaryngol*. 2022;279(7):3309-3326. doi:10.1007/s00405-021-07031-w

23. Ihler F, Volbers L, Blum J, Matthias C, Canis M. Preliminary functional results and quality of life after implantation of a new bone conduction hearing device in patients with conductive and mixed hearing loss. *Otol Neurotol*. 2014;35(2):211-215. doi:10.1097/mao.0000000000000208

24. Irmer C, Volkenstein S, Dazert S, Neumann A. The bone conduction implant BONEBRIDGE increases quality of life and social life      satisfaction. *Eur Arch Otorhinolaryngol*. 2022;279:5555-5563. doi:10.1007/s00405-022-07384-w

25. Kim Y, Choe G, Oh H, Choi BY. A comparative study of audiological outcomes and compliance between the Osia      system and other bone conduction hearing implants. *Eur Arch Otorhinolaryngol*. 2023;280:2217-2224. doi:10.1007/s00405-022-07715-x

26. Koitschev A, Neudert M, Lenarz T. A bone conduction implant using self-drilling screws. *HNO*. 2023;71(Suppl 1):61-66. doi:10.1007/s00106-023-01295-w

27. Król B, Cywka KB, Skarżyńska MB, Skarżyński PH. Implantation of the Bonebridge BCI 602 after Mastoid Obliteration with S53P4 Bioactive Glass: A Safe Method of Treating Difficult Anatomical Conditions-Preliminary Results. *Life (Basel)*. 2021;11(5). doi:10.3390/life11050374

28. Laske RD, Roosli C, Pfiffner F, Veraguth D, Huber AM. Functional Results and Subjective Benefit of a Transcutaneous Bone Conduction Device in Patients With Single-Sided Deafness. *Otol Neurotol*. 2015;36(7):1151-1156. doi:10.1097/mao.0000000000000791

29. Lassaletta L, Calvino M, Sanchez-Cuadrado I, et al. Using Generic and Disease-Specific Measures to Assess Quality of Life before and after 12 Months of Hearing Implant Use: A Prospective, Longitudinal, Multicenter, Observational Clinical Study. *Int J Environ Res Public Health*. 2022;19(5). doi:10.3390/ijerph19052503

30. Lee HJ, Kahinga AA, Moon IS. Clinical effect of an active transcutaneous bone-conduction implant on tinnitus in patients with ipsilateral sensorineural hearing loss. *Auris Nasus Larynx*. 2021;48(3):394-399. doi:10.1016/j.anl.2020.09.009

31. Lin J, Chen S, Zhang H, et al. Application of Implantable Hearing Aids and Bone Conduction Implant System in patients with bilateral congenital deformation of the external and middle ear. *Int J Pediatr Otorhinolaryngol*. 2019;119:89-95. doi:10.1016/j.ijporl.2018.12.025

32. Ngui LX, Tang IP. Bonebridge transcutaneous bone conduction implant in children with congenital      aural atresia: surgical and audiological outcomes. *J Laryngol Otol*. 2018;132:693-697. doi:10.1017/s0022215118001123

33. Oh SJ, Goh EK, Choi SW, et al. Audiologic, surgical and subjective outcomes of active transcutaneous bone conduction implant system (Bonebridge). *Int J Audiol*. 2019;58(12):956-963. doi:10.1080/14992027.2019.1657242

34. Rader T, Stover T, Lenarz T, et al. Retrospective Analysis of Hearing-Impaired Adult Patients Treated With an Active Transcutaneous Bone Conduction Implant. *Otol Neurotol*. 2018;39(7):874-881. doi:10.1097/mao.0000000000001834

35. Ratuszniak A, Skarzynski PH, Gos E, Skarzynski H. The Bonebridge implant in older children and adolescents with mixed or conductive hearing loss: Audiological outcomes. *Int J Pediatr Otorhinolaryngol*. 2019;118:97-102. doi:10.1016/j.ijporl.2018.12.026

36. Ren R, Zhao S, Wang D, et al. Audiological effectiveness of Bonebridge implantation for bilateral congenital      malformation of the external and middle ear. *Eur Arch Otorhinolaryngol*. 2019;276:2755-2762. doi:10.1007/s00405-019-05533-2

37. Salcher R, Zimmermann D, Giere T, Lenarz T, Maier H. Audiological Results in SSD With an Active Transcutaneous Bone Conduction Implant at a Retrosigmoidal Position. *Otol Neurotol*. 2017;38(5):642-647. doi:10.1097/mao.0000000000001394

38. Schmerber S, Deguine O, Marx M, et al. Safety and effectiveness of the Bonebridge transcutaneous active direct-drive bone-conduction hearing implant at 1-year device use. *Eur Arch Otorhinolaryngol*. 2017;274(4):1835-1851. doi:10.1007/s00405-016-4228-6

39. Schwam ZG, Perez ER, Oh S, et al. Initial Experience With Two Active Transcutaneous Bone-Anchored Hearing Implants. *Otol Neurotol*. 2022;43(10):1212-1215. doi:10.1097/mao.0000000000003681

40. Seiwerth I, Plößl S, Herzog M, et al. Individual computer-assisted 3D planning for placement of auricular prosthesis      anchors in combination with an implantable transcutaneous bone conduction hearing      device in patients with aural atresia. *HNO*. 2023;71:1-9. doi:10.1007/s00106-022-01190-w

41. Shapiro SB, Llerena PA, Mowery TM, Miele EA, Wackym PA. Subtemporalis Muscle Middle Cranial Fossa Bone-Island Craniotomy Technique for Placement of an Active Transcutaneous Bone-Conduction Implant. *Otol Neurotol*. 2023;44(1):54-60. doi:10.1097/mao.0000000000003760

42. Shoman NM, Khan U, Hong P. Comparison of passive versus active transcutaneous bone anchored hearing devices in the pediatric population. *J Otolaryngol Head Neck Surg*. 2022;51(1):44. doi:10.1186/s40463-022-00595-5

43. Siegel L, You P, Zimmerman K, Parnes L, Agrawal SK. Active Transcutaneous Bone Conduction Implant: Audiometric Outcomes Following a Novel Middle Fossa Approach With Self-Drilling Screws. *Otol Neurotol*. 2020;41(5):605-613. doi:10.1097/mao.0000000000002597

44. Šikolová S, Urik M, Hosnova D, et al. Two Bonebridge bone conduction hearing implant generations: audiological benefit and quality of hearing in children. *Eur Arch Otorhinolaryngol*. 2022;279(7):3387-3398. doi:10.1007/s00405-021-07068-x

45. Skarzynski PH, Krol B, Skarzynski H, Cywka KB. Implantation of two generations of Bonebridge after mastoid obliteration with bioactive glass S53P4. *Am J Otolaryngol*. 2022;43(5):103601. doi:10.1016/j.amjoto.2022.103601

46. Skarzynski PH, Ratuszniak A, Krol B, et al. The Bonebridge in Adults with Mixed and Conductive Hearing Loss: Audiological and Quality of Life Outcomes. *Audiol Neurootol*. 2019;24(2):90-99. doi:10.1159/000499363

47. Spiegel JL, Weiss BG, Bertlich M, Stoycheva I, Canis M, Ihler F. Functional results with active middle ear implant or semi-implantable bone conduction device in patients with comparable hearing loss. *Int J Audiol*. 2022;61(10):859-867. doi:10.1080/14992027.2021.1966704

48. Sprinzl G, Lenarz T, Ernst A, et al. First European multicenter results with a new transcutaneous bone conduction hearing implant system: short-term safety and efficacy. *Otol Neurotol*. 2013;34(6):1076-1083. doi:10.1097/mao.0b013e31828bb541

49. Sprinzl G, Lenarz T, Hagen R, et al. Long-Term, Multicenter Results With the First Transcutaneous Bone Conduction Implant. *Otol Neurotol*. 2021;42(6):858-866. doi:10.1097/mao.0000000000003159

50. Sprinzl G, Toner J, Koitschev A, et al. Multicentric study on surgical information and early safety and performance results with the Bonebridge BCI 602: an active transcutaneous bone conduction hearing implant. *Eur Arch Otorhinolaryngol*. 2023;280(4):1565-1579. doi:10.1007/s00405-022-07792-y

51. Sprinzl GM, Schoerg P, Ploder M, Edlinger SH, Magele A. Surgical Experience and Early Audiological Outcomes With New Active Transcutaneous Bone Conduction Implant. *Otol Neurotol*. 2021;42(8):1208-1215. doi:10.1097/mao.0000000000003230

52. Tang IP, Ling XN, Prepageran N. A review of surgical and audiological outcomes of bonebridge at tertiary centres      in Malaysia. *Med J Malaysia*. 2018;73:276-280.

53. Wang D, Ren R, Chen P, et al. Application of retrosigmoid sinus approach in Bonebridge implantation. *Acta Otolaryngol*. 2021;141(2):129-134. doi:10.1080/00016489.2020.1832702

54. Weiss R, Leinung M, Baumann U, Weissgerber T, Rader T, Stover T. Improvement of speech perception in quiet and in noise without decreasing localization abilities with the bone conduction device Bonebridge. *Eur Arch Otorhinolaryngol*. 2017;274(5):2107-2115. doi:10.1007/s00405-016-4434-2

55. Wimmer W, Werdt M von, Mantokoudis G, Anschuetz L, Kompis M, Caversaccio M. Outcome prediction for Bonebridge candidates based on audiological indication criteria. *Auris Nasus Larynx*. 2019;46(5):681-686. doi:10.1016/j.anl.2018.12.012

56. Wimmer W, Gerber N, Guignard J, et al. Topographic bone thickness maps for Bonebridge implantations. *Eur Arch Otorhinolaryngol*. 2015;272(7):1651-1658. doi:10.1007/s00405-014-2976-8

57. Yang J, Chen P, Zhao C, et al. Audiological and subjective outcomes of 100 implanted transcutaneous bone conduction devices and preoperative bone conduction hearing aids in patients with bilateral microtia-atresia. *Acta Otolaryngol*. 2020;140(8):675-681. doi:10.1080/00016489.2020.1762929

58. Yang J, Wang Z, Huang M, et al. BoneBridge implantation in patients with single-sided deafness resulting from vestibular schwannoma resection: objective and subjective benefit evaluations. *Acta Otolaryngol*. 2018;138(10):877-885. doi:10.1080/00016489.2018.1469789

59. You P, Siegel LH, Kassam Z, et al. The middle fossa approach with self-drilling screws: a novel technique for BONEBRIDGE implantation. *J Otolaryngol Head Neck Surg*. 2019;48(1):35. doi:10.1186/s40463-019-0354-7

60. Zernotti ME, Gregorio MFD, Zernotti M. Alternative Inverted Middle Fossa Approach in Bonebridge Surgery. Technique, Results and Complications. *Int Arch Otorhinolaryngol*. 2021;25(3):e374-e378. doi:10.1055/s-0040-1715152

61. Zernotti ME, Chiaraviglio MM, Mauricio SB, Tabernero PA, Zernotti M, Gregorio MFD. Audiological outcomes in patients with congenital aural atresia implanted with transcutaneous active bone conduction hearing implant. *Int J Pediatr Otorhinolaryngol*. 2019;119:54-58. doi:10.1016/j.ijporl.2019.01.016

62. Zernotti ME, Gregorio MFD, Galeazzi P, Tabernero P. Comparative outcomes of active and passive hearing devices by transcutaneous bone conduction. *Acta Otolaryngol*. 2016;136(6):556-558. doi:10.3109/00016489.2016.1143119

63. Zhao C, Yang J, Liu Y, et al. Horizontal sound localisation and speech perception in Bonebridge-implanted      single-sided deafness patients. *J Laryngol Otol*. Published online 2020:1-8. doi:10.1017/s0022215120001899

64. Alnoury MK, Daniel SJ. Minimally Invasive OSIA Bone Conduction Hearing Implant (MOSIA) in Children: How      I do it? *Laryngoscope*. 2024;134:1901-1906. doi:10.1002/lary.31001

65. Briggs R, Birman CS, Baulderstone N, et al. Clinical Performance, Safety, and Patient-Reported Outcomes of an Active      Osseointegrated Steady-State Implant System. *Otol Neurotol*. 2022;43:827-834. doi:10.1097/mao.0000000000003590

66. Florentine MM, Virbalas J, Chan DK. Early surgical and audiologic outcomes of active, transcutaneous, osseointegrated      bone-conduction hearing device (Osia 2® system) placement. *Int J Pediatr Otorhinolaryngol*. 2022;156:111114. doi:10.1016/j.ijporl.2022.111114

67. Goycoolea M, Ribalta G, Tocornal F, et al. Clinical performance of the Osia^TM^ system, a new active osseointegrated implant      system. Results from a prospective clinical investigation. *Acta Otolaryngol*. 2020;140:212-219. doi:10.1080/00016489.2019.1691744

68. Hwa TP, Locketz G, Ruckenstein MJ. Novel Surgical Technique in Active Bone Conduction: Minimally Invasive Approach to Fully Implantable Osseointegrated Implant. *Otolaryngol Head Neck Surg*. Published online 2021:1945998211044408. doi:10.1177/01945998211044408

69. Lau K, Scotta G, Wright K, et al. First United Kingdom experience of the novel Osia active transcutaneous      piezoelectric bone conduction implant. *Eur Arch Otorhinolaryngol*. 2020;277:2995-3002. doi:10.1007/s00405-020-06022-7

70. Mylanus EAM, Hua H, Wigren S, et al. Multicenter Clinical Investigation of a New Active Osseointegrated Steady-State Implant System. *Otol Neurotol*. 2020;41(9):1249-1257. doi:10.1097/mao.0000000000002794

71. Quimby AE, Park J, Brant JA, et al. Audiometric and Surgical Outcomes of a Novel Bone-Conduction Hearing Aid. *Otol Neurotol*. 2022;43(9):995-999. doi:10.1097/mao.0000000000003668

72. Rauch AK, Wesarg T, Aschendorff A, Speck I, Arndt S. Long-term data of the new transcutaneous partially implantable bone conduction      hearing system Osia®. *Eur Arch Otorhinolaryngol*. 2022;279:4279-4288. doi:10.1007/s00405-021-07167-9

73. Szabo L, Nagy R, Perenyi A, et al. Baha Attract-to-Osia conversion patients: comparison of the two systems and long term outcomes. *J Laryngol Otol*. Published online 2022:1-22. doi:10.1017/s0022215122001839

74. You P, Choi A, Drob J, Hunsaker SM, Liu YC, Silva R. Early Outcomes of a New Active Transcutaneous Bone Conduction Implant in Pediatric Patients. *Otol Neurotol*. 2022;43(2):212-218. doi:10.1097/mao.0000000000003426

75. Baker S, Centric A, Chennupati SK. Innovation in abutment-free bone-anchored hearing devices in children: Updated      results and experience. *Int J Pediatr Otorhinolaryngol*. 2015;79:1667-1672. doi:10.1016/j.ijporl.2015.07.021

76. Besten CA den, Monksfield P, Bosman A, et al. Audiological and clinical outcomes of a transcutaneous bone conduction hearing      implant: Six-month results from a multicentre study. *Clin Otolaryngol*. 2019;44:144-157. doi:10.1111/coa.13248

77. Briggs R, Hasselt AV, Luntz M, et al. Clinical performance of a new magnetic bone conduction hearing implant system:      results from a prospective, multicenter, clinical investigation. *Otol Neurotol*. 2015;36:834-841. doi:10.1097/mao.0000000000000712

78. Denoyelle F, Coudert C, Thierry B, et al. Hearing rehabilitation with the closed skin bone-anchored implant Sophono Alpha1:      results of a prospective study in 15 children with ear atresia. *Int J Pediatr Otorhinolaryngol*. 2015;79:382-387. doi:10.1016/j.ijporl.2014.12.032

79. Dimitriadis PA, Hind D, Wright K, et al. Single-center Experience of Over a Hundred Implantations of a Transcutaneous Bone      Conduction Device. *Otol Neurotol*. 2017;38:1301-1307. doi:10.1097/mao.0000000000001529

80. Dolhen P, Lipski S, Touijar R, Bogaert JV. Minimal invasive pocket technique for magnet bone implant hearing aid without      fixation. *Eur Arch Otorhinolaryngol*. 2020;277:715-725. doi:10.1007/s00405-019-05746-5

81. Gawecki W, Balcerowiak A, Kalinowicz E, Wrobel M. Evaluation of surgery and surgical results of Baha((R)) Attract system implantations - single centre experience of hundred twenty five cases. *Braz J Otorhinolaryngol*. 2019;85(5):597-602. doi:10.1016/j.bjorl.2018.04.011

82. Giannantonio S, Scorpecci A, Pacifico C, Marsella P. A functional and anatomical comparison between two passive transcutaneous bone      conduction implants in children. *Int J Pediatr Otorhinolaryngol*. 2018;108:202-207. doi:10.1016/j.ijporl.2018.03.007

83. Godbehere J, Carr SD, Moraleda J, Edwards P, Ray J. A comparison study of complications and initial follow-up costs of transcutaneous and percutaneous bone conduction devices. *J Laryngology X0026 Otology*. 2017;131(8):667-670. doi:10.1017/s002221511700127x

84. Hougaard DD, Boldsen SK, Jensen AM, Hansen S, Thomassen PC. A multicenter study on objective and subjective benefits with a transcutaneous      bone-anchored hearing aid device: first Nordic results. *Eur Arch Otorhinolaryngol*. 2017;274:3011-3019. doi:10.1007/s00405-017-4614-8

85. Iseri M, Orhan KS, Tuncer U, et al. Transcutaneous Bone-anchored Hearing Aids Versus Percutaneous Ones. *Otol Neurotol*. 2015;36(5):849-853. doi:10.1097/mao.0000000000000733

86. Leterme G, Bernardeschi D, Bensemman A, et al. Contralateral routing of signal hearing aid versus transcutaneous bone conduction      in single-sided deafness. *Audiol Neurootol*. 2015;20:251-260. doi:10.1159/000381329

87. Kruyt IJ, Monksfield P, Skarzynski PH, et al. Results of a 2-Year Prospective Multicenter Study Evaluating Long-term Audiological and Clinical Outcomes of a Transcutaneous Implant for Bone Conduction Hearing. *Otol Neurotol*. 2020;41(7):901-911. doi:10.1097/mao.0000000000002689

88. Luque CG, Liu AQ, Pauwels J, Leitmeyer K, Chadha NK. Patient reported outcome measures in unilateral aural atresia treated using a      transcutaneous bone conduction implant (The Cochlear Baha Attract®). *Int J Pediatr Otorhinolaryngol*. 2023;165:111451. doi:10.1016/j.ijporl.2023.111451

89. Magliulo G, Iannella G, Vincentiis MD, et al. Transcutaneous bone conductive implants in patients with conductive/mixed hearing      loss: audiological outcomes in noise condition. *Acta Otolaryngol*. 2018;138:822-829. doi:10.1080/00016489.2018.1478128

90. Nevoux J, Coudert C, Boulet M, et al. Transcutaneous Baha Attract system: Long-term outcomes of the French multicenter      study. *Clin Otolaryngol*. 2018;43:1553-1559. doi:10.1111/coa.13214

91. Oberlies NR, Castano JE, Freiser ME, McCoy JL, Shaffer AD, Jabbour N. Outcomes of BAHA connect vs BAHA attract in pediatric patients. *Int J Pediatr Otorhinolaryngol*. 2020;135:110125. doi:10.1016/j.ijporl.2020.110125

92. Pérez-Carbonell T, Pla-Gil I, Redondo-Martínez J, Morant-Ventura A, García-Callejo FJ, Marco-Algarra J. Audiologic and Subjective Evaluation of Baha® Attract Device. *Acta Otorrinolaringologica Engl Ed*. 2017;68(6):344-348. doi:10.1016/j.otoeng.2017.10.004

93. Plasencia DP, Suárez AA, Barreiro SB, Miguel AR de, Macias AR. Transcutaneous Osseo-integrated Auditory Devices: Analysis of Two Different Implants in Adults With Different Audiological Inclusion Criteria. *Otol Neurotol*. 2021;42(9):e1308-e1312. doi:10.1097/mao.0000000000003286

94. Powell HRF, Rolfe AM, Birman CS. A Comparative Study of Audiologic Outcomes for Two Transcutaneous Bone-Anchored      Hearing Devices. *Otol Neurotol*. 2015;36:1525-1531. doi:10.1097/mao.0000000000000842

95. Sharma S, Reddy‐Kolanu G, Marshall AH. UK tertiary centre experience of outcomes from osseointegrated transcutaneous magnetic bone conduction hearing system implanted in twenty‐five patients using a linear incision technique. *Clin Otolaryngol*. 2017;42(5):1041-1043. doi:10.1111/coa.12815

96. Shokri T, Czarnecki B, Baker A, Isildak H. Hearing Rehabilitation Implementing a Transcutaneous Bone Conduction Device:      Single-Center Experience. *Ear Nose Throat J*. 2021;100:199S-203S. doi:10.1177/0145561319870481

97. Aldhafeeri AM, Yousef M, Alzhrani F. Osseointegrated device placement with minimally invasive surgery: Experience and      audiological outcome. *Saudi Med J*. 2022;43:530-533. doi:10.15537/smj.2022.43.5.20210913

98. Allis TJ, Owen BD, Chen B, Jones DT, Moore GF. Longer length baha^TM^ abutments decrease wound complications and revision surgery. *Laryngoscope*. 2014;124(4):989-992. doi:10.1002/lary.24399

99. Amaral MSA do, Santos FRD, Danieli F, Massuda ET, Reis ACMB, Hyppolito MA. Surgical and audiological results of bone-anchored hearing aids: comparison of      two surgical techniques. *Braz J Otorhinolaryngol*. 2022;88:533-538. doi:10.1016/j.bjorl.2020.07.003

100. Amin N, Soulby AJ, Borsetto D, Pai I. Longitudinal economic analysis of Bonebridge 601 versus percutaneous      bone-anchored hearing devices over a 5-year follow-up period. *Clin Otolaryngol*. 2021;46:263-272. doi:10.1111/coa.13659

101. Amonoo-Kuofi K, Kelly A, Neeff M, Brown CRS. Experience of bone-anchored hearing aid implantation in children younger than 5 years of age. *Int J Pediatr Otorhinolaryngol*. 2015;79(4):474-480. doi:10.1016/j.ijporl.2014.12.033

102. Asma A, Ubaidah MA, Hasan SS, et al. Surgical Outcome of Bone Anchored Hearing Aid (Baha) Implant Surgery: A 10 Years Experience. *Indian J Otolaryngol*. 2013;65(3):251-254. doi:10.1007/s12070-013-0621-2

103. Besten CA den, Harterink E, McDermott AL, Hol MKS. Clinical results of Cochlear^TM^ BIA300 in children: Experience in two tertiary referral centers. *Int J Pediatr Otorhi*. 2015;79(12):2050-2055. doi:10.1016/j.ijporl.2015.09.010

104. Besten CA den, Bosman AJ, Nelissen RC, Mylanus EAM, Hol MKS. Controlled Clinical Trial on Bone-anchored Hearing Implants and a Surgical Technique With Soft-tissue Preservation. *Otol Neurotol*. 2016;37(5):504-512. doi:10.1097/mao.0000000000000994

105. Bonilla A, Magri C, Juan E. Findings from the experience with the punch technique for auditory osseointegrated implants: A retrospective single center comparative study. *Acta Otorrinolaringológica Española*. 2017;68(6):309-316. doi:10.1016/j.otorri.2017.01.005

106. Bouček J, Vokřál J, Černý L, et al. Baha implant as a hearing solution for single-sided deafness after retrosigmoid      approach for the vestibular schwannoma: audiological results. *Eur Arch Otorhinolaryngol*. 2017;274:133-141. doi:10.1007/s00405-016-4261-5

107. Brant JA, Gudis D, Ruckenstein MJ. Results of Baha® implantation using a small horizontal incision. *Am J Otolaryng*. 2013;34(6):641-645. doi:10.1016/j.amjoto.2013.07.005

108. Calon TGA, Tongeren J van, Omar O, Johansson ML, Stokroos RJ. Cytokine expression profile in the bone-anchored hearing system: 12-week results      from a prospective randomized, controlled study. *Clin Implant Dent Relat Res*. 2018;20:606-616. doi:10.1111/cid.12615

109. Calon TGA, Trobos M, Johansson ML, et al. Microbiome on the Bone-Anchored Hearing System: A Prospective Study. *Front Microbiol*. 2019;10:799. doi:10.3389/fmicb.2019.00799

110. Candreia C, Birrer R, Fistarol S, et al. Predisposing factors for adverse skin reactions with percutaneous bone anchored hearing devices implanted with skin reduction techniques. *Eur Arch Oto-rhino-l*. 2016;273(12):4185-4192. doi:10.1007/s00405-016-4106-2

111. Carr SD, Moraleda J, Baldwin A, Ray J. Bone-conduction hearing aids in an elderly population: complications and quality of life assessment. *Eur Arch Oto-rhino-l*. 2016;273(3):567-571. doi:10.1007/s00405-015-3574-0

112. Caspers CJI, Kruyt IJ, Mylanus EAM, Hol MKS. A Clinical Evaluation of Minimally Invasive Ponto Surgery With a Modified Drill System for Inserting Bone-Anchored Hearing Implants. *Otol Neurotol*. 2021;42(8):1192-1200. doi:10.1097/mao.0000000000003195

113. Costa JR, Costa S, Soares T, Feliciano T, Sousa CAE, Coutinho MB. Skin and soft tissue complications of bone-anchored hearing aids: Introducing a      new classification system. *Acta Otorrinolaringol Esp (Engl Ed)*. 2023;74:85-92. doi:10.1016/j.otoeng.2022.01.004

114. Cruz LDS, Danieli F, Håkansson MÅ, et al. Minimally invasive surgery as a new clinical standard for bone anchored hearing      implants-real-world data from 10 years of follow-up and 228 surgeries. *Front Surg*. 2023;10:1209927. doi:10.3389/fsurg.2023.1209927

115. Cuda D, Murri A, Mochi P, Mainardi A. Postoperative Benefit of Bone Anchored Hearing Systems: Behavioral Performance and Self-Reported Outcomes. *Int Arch Otorhinolaryngol*. 2022;26(3):e314-e320. doi:10.1055/s-0040-1718959

116. D’Eredita R, Caroncini M, Saetti R. The new Baha implant: a prospective osseointegration study. *Otolaryngol Head Neck Surg*. 2012;146(6):979-983. doi:10.1177/0194599812438042

117. Darley MD, Mikulec AA. Survival of the 8.5 mm osseointegrated abutment, and its utility in the obese patient. *J Laryngology Otology*. 2013;127(7):643-649. doi:10.1017/s0022215113001072

118. Dawe N, Leese D, Marley S, McPherson K, Johnson IJM. The use of bone conduction hearing implants in paediatric chronic otitis media: An audit of outcomes of 32 devices in 22 patients. *Clin Otolaryngol*. 2019;44(1):83-86. doi:10.1111/coa.13235

119. Doshi J, Banga R, Child A, et al. Quality-of-Life Outcomes After Bone-Anchored Hearing Device Surgery in Children With Single-Sided Sensorineural Deafness. *Otol Neurotol*. 2013;34(1):100-103. doi:10.1097/mao.0b013e318277a3dd

120. Faber HT, Dun CAJ, Nelissen RC, Mylanus EAM, Cremers CWRJ, Hol MKS. Bone-Anchored Hearing Implant Loading at 3 Weeks. *Otol Neurotol*. 2013;34(1):104-110. doi:10.1097/mao.0b013e318277a282

121. Dumon T, Medina M, Sperling NM. Punch and Drill: Implantation of Bone Anchored Hearing Device Through a Minimal      Skin Punch Incision Versus Implantation With Dermatome and Soft Tissue Reduction. *Ann Otol Rhinol Laryngol*. 2016;125:199-206. doi:10.1177/0003489415606447

122. Farnoosh S, Mitsinikos FT, Maceri D, Don DM. Bone-Anchored Hearing Aid vs. Reconstruction of the External Auditory Canal in Children and Adolescents with Congenital Aural Atresia: A Comparison Study of Outcomes. *Frontiers Pediatrics*. 2014;2:5. doi:10.3389/fped.2014.00005

123. Foghsgaard S, Caye-Thomasen P. A new wide-diameter bone-anchored hearing implant-prospective 1-year data on complications, implant stability, and survival. *Otol Neurotol*. 2014;35(7):1238-1241. doi:10.1097/mao.0000000000000345

124. Fontaine N, Hemar P, Schultz P, Charpiot A, Debry C. BAHA implant: Implantation technique and complications. *European Ann Otorhinolaryngology Head Neck Dis*. 2014;131(1):69-74. doi:10.1016/j.anorl.2012.10.006

125. French G, Lee KH, Yunker WK. A retrospective evaluation of minimally invasive ponto surgery (MIPS) in two pediatric centers. *Cochlear Implants Int*. 2021;22(5):1-5. doi:10.1080/14670100.2021.1903712

126. Fussey JM, Harterink E, Gill J, Child-Hymas A, McDermott A louise. Clinical outcomes following Cochlear^TM^ BIA300 bone anchored hearing aid implantation in children. *Int J Pediatr Otorhinolaryngol*. 2018;111:89-92. doi:10.1016/j.ijporl.2018.05.033

127. Gaskell P, Muzaffar J, Kumar R, Limbrick J, Monksfield P, Banga R. Surgical outcomes with the tissue preservation technique in bone-anchored hearing implants: The Birmingham experience of ninety-seven cases. *Clin Otolaryngol*. 2021;46(4):846-849. doi:10.1111/coa.13728

128. Hernández S, Ospina JC, Gutiérrez-Gómez E, Rodríguez-Ruiz MT, Escobar JL. Long term cutaneous complications related to bone conduction hearing implants. A retrospective study (2004-2018). *Auris Nasus Larynx*. 2021;48(5):878-884. doi:10.1016/j.anl.2021.01.020

129. Hogsbro M, Agger A, Johansen LV. Bone Anchored Hearing Implant Surgery: 1 Year Follow-Up Data Shows No Effect of Hydroxyapatite Coating on Soft Tissue Reaction After Loading at 1 Week. *Otol Neurotol*. 2017;38(6):e152-e158. doi:10.1097/mao.0000000000001442

130. Høgsbro M, Agger A, Johansen LV. Successful Loading of a Bone-Anchored Hearing Implant at Two Weeks After Surgery. *Otol Neurotol*. 2015;36(2):e51-e57. doi:10.1097/mao.0000000000000647

131. Holmes S, Hamiter M, Berry C, Mankekar G. Tissue Preservation Techniques for Bone-Anchored Hearing Aid Surgery. *Otol Neurotol*. 2021;42:1044-1050. doi:10.1097/mao.0000000000003157

132. Hoof M van, Wigren S, Blechert JI, et al. Clinical Outcomes of Soft Tissue Preservation Surgery With Hydroxyapatite-Coated      Abutments Compared to Traditional Percutaneous Bone Conduction Hearing Implant      Surgery-A Pragmatic Multi-Center Randomized Controlled Trial. *Front Surg*. 2020;7:5. doi:10.3389/fsurg.2020.00005

133. Hultcrantz M, Lanis A. Prospective analysis of stability testing for bone-anchored hearing implants in children after osseointegrating surgery without skin thinning. *Int J Pediatr Otorhi*. 2015;79(4):465-468. doi:10.1016/j.ijporl.2014.12.019

134. Johansson ML, Stokroos RJ, Banga R, et al. Short-term results from seventy-six patients receiving a bone-anchored hearing implant installed with a novel minimally invasive surgery technique. *Clin Otolaryngol*. 2017;42(5):1043-1048. doi:10.1111/coa.12803

135. Kanzara T, Walijee H, Sheikh RB, Lau A, Temple R. Long-term soft tissue outcomes for hydroxyapatite-coated bone-anchored hearing implant surgery. *Eur Arch Otorhinolaryngol*. 2019;276(11):3067-3072. doi:10.1007/s00405-019-05609-z

136. Kim HHS, Kari E, Copeland BJ, et al. Standardization of the Punch Technique for the Implantation of Bone Anchored      Auditory Devices: Evaluation of the MIPS Surgical Set. *Otol Neurotol*. 2019;40:e631-e635. doi:10.1097/mao.0000000000002291

137. King J, Leon I, Squires L. In-office Bone-Anchored Hearing Implants via Minimally Invasive Punch Technique in a Veteran Population. *Otolaryngol Head Neck Surg*. 2022;167(6):959-963. doi:10.1177/01945998221086841

138. Kruyt IJ, Banga R, Banerjee A, Mylanus EAM, Hol MKS. Clinical evaluation of a new laser-ablated titanium implant for bone-anchored hearing in 34 patients: 1-year experience. *Clin Otolaryngol*. 2018;43(2):761-764. doi:10.1111/coa.13060

139. Kruyt IJ, Kok H, Bosman A, Nelissen RC, Mylanus EAM, Hol MKS. Three-Year Clinical and Audiological Outcomes of Percutaneous Implants for Bone Conduction Devices: Comparison Between Tissue Preservation Technique and Tissue Reduction Technique. *Otol Neurotol*. 2019;40(3):335-343. doi:10.1097/mao.0000000000002105

140. Lanis A, Hultcrantz M. Percutaneous Osseointegrated Implant Surgery Without Skin Thinning in Children. *Otol Neurotol*. 2013;34(4):715-722. doi:10.1097/mao.0b013e31827de4dd

141. Marsella P, Scorpecci A, D’Eredità R, Volpe AD, Malerba P. Stability of Osseointegrated Bone Conduction Systems in Children. *Otol Neurotol*. 2012;33(5):797-803. doi:10.1097/mao.0b013e318255dd73

142. McElveen JTJ, Green JDJ, Arriaga MA, Slattery WH 3rd. Next-Day Loading of a Bone-Anchored Hearing System: Preliminary Results. *Otolaryngol Head Neck Surg*. 2020;163:582-587. doi:10.1177/0194599820915465

143. McLarnon CM, Johnson I, Davison T, et al. Evidence for Early Loading of Osseointegrated Implants for Bone Conduction at 4 Weeks. *Otol Neurotol*. 2012;33(9):1578-1582. doi:10.1097/mao.0b013e31826dba5f

144. Mowinckel MS, Møller MN, Wielandt KN, Foghsgaard S. Clinical Outcome of a Wide-diameter Bone-anchored Hearing Implant and a Surgical Technique With Tissue Preservation. *Otol Neurotol*. 2016;37(4):374-379. doi:10.1097/mao.0000000000000990

145. Nelissen RC, Mylanus EAM, Kunst HPM, Pennings RJE, Snik AFM, Hol MKS. A new bone-anchored hearing implant: short-term retrospective data on implant      survival and subjective benefit. *Eur Arch Otorhinolaryngol*. 2013;270:3019-3025. doi:10.1007/s00405-013-2346-y

146. Nelissen RC, Besten CA den, Faber HT, Dun CAJ, Mylanus EAM, Hol MKS. Loading of osseointegrated implants for bone conduction hearing at 3 weeks: 3-year stability, survival, and tolerability. *Eur Arch Oto-rhino-l*. 2016;273(7):1731-1737. doi:10.1007/s00405-015-3746-y

147. Nelissen RC, Besten CA den, Mylanus EAM, Hol MKS. Stability, survival, and tolerability of a 4.5-mm-wide bone-anchored hearing      implant: 6-month data from a randomized controlled clinical trial. *Eur Arch Otorhinolaryngol*. 2016;273:105-111. doi:10.1007/s00405-015-3593-x

148. Nelissen RC, Agterberg MJH, Hol MKS, Snik AFM. Three-year experience with the Sophono in children with congenital conductive      unilateral hearing loss: tolerability, audiometry, and sound localization      compared to a bone-anchored hearing aid. *Eur Arch Otorhinolaryngol*. 2016;273:3149-3156. doi:10.1007/s00405-016-3908-6

149. Nelson KL, Cox MD, Richter GT, Dornhoffer JL. A Comparative Review of Osseointegration Failure Between Osseointegrated Bone Conduction Device Models in Pediatric Patients. *Otol Neurotol*. 2016;37(3):276-280. doi:10.1097/mao.0000000000000970

150. Osborne MS, Child-Hymas A, McDermott AL. Clinical evaluation and resonance frequency analysis of laser-ablated titanium      bone-anchored hearing implant system in children with Down Syndrome. *Int J Pediatr Otorhinolaryngol*. 2021;151:110981. doi:10.1016/j.ijporl.2021.110981

151. Osborne MS, Child-Hymas A, Holmberg M, Thomsen P, Johansson ML, McDermott AL. Clinical Evaluation of a Novel Laser-Ablated Titanium Implant System for Bone Anchored Hearing Systems in a Pediatric Population and the Relationship of Resonance Frequency Analysis With Implant Survival. *Otol Neurotol*. 2022;43(2):219-226. doi:10.1097/mao.0000000000003435

152. Osborne MS, Hoskison E, Child-Hymas A, Gill J, McDermott AL. Five year clinical outcomes and evaluation following implantation of the Oticon^TM^ wide bone anchored hearing system in 47 children. *Int J Pediatr Otorhinolaryngol*. 2020;137:110244. doi:10.1016/j.ijporl.2020.110244

153. Peters JPM, Heteren JAA van, Wendrich AW, et al. Short-term outcomes of cochlear implantation for single-sided deafness compared to bone conduction devices and contralateral routing of sound hearing aids—Results of a Randomised controlled trial (CINGLE-trial). *Plos One*. 2021;16(10):e0257447. doi:10.1371/journal.pone.0257447

154. Ray J, Addams-Williams J, Baldwin A. Minimal Access Surgery for Implantable Bone Conduction Systems. *Otol Neurotol*. 2012;33(7):1232-1234. doi:10.1097/mao.0b013e318263d780

155. Ray J, Lau K, Moraleda J, Yardley M, Dawoud M, Dimitriadis PA. Soft-tissue outcomes following implantation of different types of bone conduction hearing devices in a single centre. *J Laryngology Otology*. 2019;133(12):1079-1082. doi:10.1017/s002221511900241x

156. Reznitsky M, Wielandt K, Foghsgaard S. Wide diameter bone-anchored hearing system implants: a comparison of long-term      follow-up data between tissue reduction and tissue preservation techniques. *Eur Arch Otorhinolaryngol*. 2019;276:349-356. doi:10.1007/s00405-018-5228-5

157. Roplekar R, Lim A, Hussain SSM. Has the use of the linear incision reduced skin complications in bone-anchored hearing aid implantation? *J Laryngology X0026 Otology*. 2016;130(6):541-544. doi:10.1017/s0022215116001080

158. Shin JW, Kim SH, Choi JY, et al. Surgical and Audiologic Comparison Between Sophono and Bone-Anchored Hearing Aids      Implantation. *Clin Exp Otorhinolaryngol*. 2016;9:21-26. doi:10.21053/ceo.2016.9.1.21

159. Shoman NM. Single-Center Experience Evaluating Clinical Outcomes of a Novel-Guided Drill System for Percutaneous Bone-Anchored Hearing Implant. *Otol Neurotol*. 2022;43(8):e797-e803. doi:10.1097/mao.0000000000003634

160. Skarzynski PH, Dziendziel B, Wlodarczyk E, Skarzynski H. The Oticon Ponto System in Adults With Severe-to-Profound and Mixed Hearing Loss:      Audiologic Outcomes and Patient Satisfaction. *Otol Neurotol*. 2022;43:987-994. doi:10.1097/mao.0000000000003664

161. Strijbos RM, Straatman LV, Calon TGA, et al. Long-Term Outcomes of the Minimally Invasive Ponto Surgery vs. Linear Incision      Technique With Soft Tissue Preservation for Installation of Percutaneous Bone      Conduction Devices. *Front Neurol*. 2021;12:632987. doi:10.3389/fneur.2021.632987

162. Strijbos RM, Salameh S, Bezdjian A, Daniel SJ, Thomeer HG. The Minimally Invasive Star-Shaped Incision Technique and the Linear Incision      Technique With Tissue Preservation for Percutaneous Bone Conduction Devices: A      Retrospective Cohort Study. *Front Surg*. 2022;9:863997. doi:10.3389/fsurg.2022.863997

163. Succar ACS, Sassi TS da S, Neto RV de B, Lourençone LFM. Complications and audiological results of percutaneous bone-anchored hearing      devices. *J Laryngol Otol*. 2024;138:391-397. doi:10.1017/s0022215123001251

164. Tjellström A, Stalfors J. Bone-anchored hearing device surgery: a 3- to 6-year follow-up with life table and worst-case scenario calculation. *Otol Neurotol : Off Publ Am Otol Soc, Am Neurotol Soc Eur Acad Otol Neurotol*. 2012;33(5):891-894. doi:10.1097/mao.0b013e3182565b2e

165. Topcu MT, Mutlu B, Celik S, Celikgun B, Mutlu A, Kalcioglu MT. Bone-Anchored Hearing Implants: Surgical and Audiological Comparison of Different Surgical Techniques. *Int Arch Otorhinolaryngol*. 2022;26(4):e649-e656. doi:10.1055/s-0042-1742765

166. Trobos M, Johansson ML, Jonhede S, et al. The clinical outcome and microbiological profile of bone-anchored hearing systems (BAHS) with different abutment topographies: a prospective pilot study. *Eur Arch Otorhinolaryngol*. 2018;275(6):1395-1408. doi:10.1007/s00405-018-4946-z

167. Wazen JJ, Babu S, Daugherty J, Metrailer A. Three-week loading of the 4.5mm wide titanium implant in bone anchored hearing      systems. *Am J Otolaryngol*. 2016;37:132-135. doi:10.1016/j.amjoto.2015.08.005

168. Wilkie MD, Chakravarthy KM, Mamais C, Temple RH. Osseointegrated Hearing Implant Surgery Using a Novel Hydroxyapatite-Coated Concave Abutment Design. *Otolaryngology Head Neck Surg*. 2014;151(6):1014-1019. doi:10.1177/0194599814551150

169. Wilson DF, Kim HH. A minimally invasive technique for the implantation of bone-anchored hearing      devices. *Otolaryngol Head Neck Surg*. 2013;149:473-477. doi:10.1177/0194599813492946

170. Van der Gucht K, Vanderveken O, Hamans E, Claes J, Van Rompaey V, Van de Heyning P. Adverse skin reactions following percutaneous bone conduction implant surgery using the linear incision technique with and without subcutaneous tissue reduction. *Acta Oto-laryngol*. 2017;137(2):149-153. doi:10.1080/00016489.2016.1222548
